# Trends of research productivity across author gender and research fields: A multidisciplinary and multi-country observational study

**DOI:** 10.1371/journal.pone.0271998

**Published:** 2022-08-10

**Authors:** Milad Haghani, Alireza Abbasi, Clara C. Zwack, Zahra Shahhoseini, Nick Haslam

**Affiliations:** 1 Research Centre for Integrated Transport Innovation (rCITI), School of Civil and Environmental Engineering, The University of New South Wales, UNSW, Sydney, Australia; 2 School of Engineering and Information Technology (SEIT), UNSW, Canberra, Australia; 3 Sydney School of Health Sciences, Faculty of Medicine and Health, The University of Sydney, Sydney, Australia; 4 Level Crossing Removal Projects, Major Transport Infrastructure Authority, Melbourne, Australia; 5 Melbourne School of Psychological Sciences, The University of Melbourne, Melbourne, Australia; Oxford University Hospitals NHS Foundation Trust, UNITED KINGDOM

## Abstract

Bibliographic properties of more than 75 million scholarly articles, are examined and trends in overall research productivity are analysed as a function of research field (over the period of 1970–2020) and author gender (over the period of 2006–2020). Potential disruptive effects of the Covid-19 pandemic are also investigated. Over the last decade (2010–2020), the annual number of publications have invariably increased every year with the largest relative increase in a single year happening in 2019 (more than 6% relative growth). But this momentum was interrupted in 2020. Trends show that Environmental Sciences and Engineering Environmental have been the fastest growing research fields. The disruption in patterns of scholarly publication due to the Covid-19 pandemic was unevenly distributed across fields, with Computer Science, Engineering and Social Science enduring the most notable declines. The overall trends of male and female productivity indicate that, in terms of absolute number of publications, the gender gap does not seem to be closing in any country. The trends in absolute gap between male and female authors is either parallel (e.g., Canada, Australia, England, USA) or widening (e.g., majority of countries, particularly Middle Eastern countries). In terms of the ratio of female to male productivity, however, the gap is narrowing almost invariably, though at markedly different rates across countries. While some countries are nearing a ratio of .7 and are well on track for a 0.9 female to male productivity ratio, our estimates show that certain countries (particularly across the Middle East) will not reach such targets within the next 100 years. Without interventional policies, a significant gap will continue to exist in such countries. The decrease or increase in research productivity during the first year of the pandemic, in contrast to trends established before 2020, was generally parallel for male and female authors. There has been no substantial gender difference in the disruption due to the pandemic. However, opposite trends were found in a few cases. It was observed that, in some countries (e.g., The Netherlands, The United States and Germany), male productivity has been more negatively affected by the pandemic. Overall, female research productivity seems to have been more resilient to the disruptive effect of Covid-19 pandemic, although the *momentum* of female researchers has been negatively affected in a comparable manner to that of males.

## Introduction

The growth of global scholarly research activity and its dynamics have for years been a major focus of fields such as *science of science* and *information science* [1–4]. Previous studies have looked at the expansion of scholarly literature and have attempted to infer current patterns and predict future directions [5, 6]. Such information is essential for informing scientific communities and policy makers of how the landscape of research around the world is changing. Such information can help governments allocate research funding, research institutions guide hiring and promotion decisions, and individual researchers maximise the impact of their work.

While progression of scholarly research can be measured through a variety of indicators [7], a primary indicator of the growth of research has traditionally been the number of scholarly publications [2, 4, 8]. Despite the imperfectness of this metric for measuring research productivity—both at the level of individual scientists [9–11] and aggregate levels [12–14]—it has remained the primary metric for quantifying research production [8]. As Riviera [15] has put it “Communication in science is realized through publications. Thus, scientific explanations, and in general scientific knowledge, are contained in written documents constituting scientific literature” (p. 1446).

It has been established that the growth of science does not take place at an equal rate across fields [2]. The phenomenon of “field variation” or “field-dependence” [16, 17] has been document in several studies [18]. One component of our analyses is dedicated to identifying differential patterns of growth in different fields of science while also detecting potential disruptive impacts of Covid-19 pandemic on such patterns. Studies that have investigated or predicted the potential disruptive effects of Covid-19 pandemic on research productivity have mostly produced signs of negative impact [19–21]. These are predominantly based on the analysis of pre-prints of publications (or working papers). Negative impact has been documented in relation to the knowledge production of social scientists [22] and economists [23]. However, some early analyses predicted an increase in productivity in certain fields such as economics and finance [24]. It has also been argued that the pandemic will affect research fields and groups of researchers differently. Termini and Traver [19], for example, point out that “In response to the pandemic, research institutions have enacted strict changes to permitted research operations, requiring scientists to abide by social distancing guidelines in the laboratory, facility closures, and ramped down laboratory activities. While scientists at all stages in their careers have been impacted by these changes to the research environment, early career scientists such as postdoctoral fellows and junior faculty are particularly vulnerable during these unconventional times” (p1). Of particular interest in the present study is to determine which research fields experienced the largest impact of the pandemic disruption, based on publication output.

This study is also focused on determining potential discrepancies in the disruption of productivity trends across male and female scientists. The issue of gender representation in scientific publishing is perhaps one of the most documented and most heavily discussed aspects in the science of science [25–27]. Several case studies have documented the gender inequality problem across different countries such as Italy [28], Canada [29], Sweden [30], and Norway [31], to name a few. The origins of the gender gap problem have been much debated in the literature and several explanations have been offered by previous studies [32–36]. Clearly, when considering the aggregate representation of male and female researchers in publications, one main factor that explains the gap is under-representation of female scientists in academic positions [37]. Other factors have also been investigated, most important of which being motherhood and childcare responsibilities [38–41], and the disparities in the extent of opportunities for collaborations across male and female scientists [27, 38, 42].

In view of these contributors to the gender gap in research, how might the Covid-19 pandemic have had a differential impact on female and male researchers? The dynamics of family responsibilities and childcare have undergone considerable changes in 2020 [43–48] as a result of the work-from-home arrangements and/or lack of access to external childcare services in many countries. This raises the question of whether this factor has played a role in overall (aggregate) productivity of female scientists. Covid-related loss of access to facilities, mentorship and in-person meetings with peers may have exacerbated the gender gap in research productivity by reducing opportunities for collaboration. Conversely, as online meetings and webinars became more prevalent since the onset of the pandemic, more opportunities may have arisen for collaborations, especially at an international level. Although there is insufficient research evidence to date, some writers have argued that the pandemic’s negative impacts will be fall disproportionately on women. It has been argued that “The coronavirus disease 2019 (COVID-19) pandemic has upended almost every facet of academia (1). Almost overnight the system faced a sudden transition to remote teaching and learning, changes in grading systems, and the loss of access to research resources. Additionally, shifts in household labour, childcare, eldercare, and physical confinement have increased students’ and faculty’s mental health needs and reduced the time available to perform academic work…Many women academics will likely bear a greater burden during the coronavirus disease 2019 (COVID-19) pandemic” (p. 27) [49]. Similarly the findings of a preliminary survey of American and European scientists in April 2020 predicted that “female scientists and those with young dependents were to be affected disproportionately” [50]. Nearly two years since the onset of the pandemic, now there is an opportunity to objectively quantify these effects.

This study aims to examine trends in overall research productivity as well as the possible differential productivity across research fields (1970–2020) and author genders (2006–2020). While the trends in representation of male and female scholarly publication are revisited across different cultures (2006–2020), the potential disruptive impacts of Covid-19 are also quantified based on research production of male and female scientists during 2020, as compared with their respective trends. The data also provides insight into the trends in the gap between male and female representation in scholarly publications and the expected time frame for the gap to close in different world regions should existing trends continue.

## Methods and data collection

Three sets of publication meta data were collected from WoS, each using a combination of search query strings. The query strings are all formulated for the “Advanced Search” engine of the WoS Core Collection. All analyses are based on meta data of publication counts and they reflect “all document types”.

### Research fields

The first set of meta data contains records of publication counts of the top 100 WoS Categories, in terms of the volume of publications attributed to each category (out of the 254 categories recognised by the WoS). The list has been obtained by acquiring all publication records since 2000 and listing the WoS Categories in descending order based on the number of publications counts. Subsequently, 100 different search queries were formulated and entered into WoS Advanced Search. Each query reads as “WC = [the name of the category as specified by the WoS]”, where WC is the Field Tag for WoS Category. From the outcome of each query, the meta data of publication count (of all document types), during Jan 1970 till Dec 2020, were exported and stored. This time period was applied to all disciplines. While the data of each category is analysed separately, for the presentation purposes, these categories were also further categorised into “broad discipline areas”. These areas include (in alphabetical order) “Agricultural Sciences”, “Arts & Humanities, Interdisciplinary”, “Biology & Biochemistry”, “Chemistry”, “Clinical Medicine”, “Computer Science”, “Economics & Business”, “Engineering”, “Environment/Ecology”, “Geosciences”, “History & Archaeology”, “Literature & Language”, “Materials Science”, “Mathematics”, “Multidisciplinary”, “Philosophy & Religion”, “Physics”, “Plant & Animal Science”, “Psychiatry/Psychology”, “Social Sciences, General” and “Visual & Performing Arts”. Only broad areas that have at least one category in the list of top 100 get a mention in our analysis. Also, some of the WoS Categories have been attributed to more than one broad category. In such cases and for presentation purposes, we allocated the category randomly to one broad discipline.

### Author gender

Two further sets of meta data were exported for the gender analyses. Determination of the author gender has traditionally been made based on the first name of the authors. This, however, is typically done on a pre-sampled set of publications, whereas here, we sought to establish a reverse approach where a search query is used to generate samples of papers with male and female authors. In doing so, we formulated our queries based on the Author(s) search function of the WoS. According to the WoS guidelines for searching names of authors, there is no way to only search for the first name of authors. The acceptable format is AU = [“surname” SPACE “first name”] (where AU is the Field Tag), and if only one entry is specified within the search, then that is regarded as the surname by the search engine. So, the question would be; is there any way that one can generate all publications with authors of a specific first name (in any position of authorship, first, middle or last, noting that the WoS search engine does not differentiate between authorship positions). It was discovered that this can be done through the asterisk (*) wildcard. Consider the query AU = A* Albert. Such query would return any publication on which an author with first name of “Albert” and a surname with the initial “A” is listed. When the query is extended to AU = (A* Albert OR B* Albert OR C* Albert OR … OR Z* Albert) (where OR is a Boolean operator), it generates all documents on which at least one Albert is listed as an author. This method constitutes the essence of our proposed search strategy.

Assume that one develops a query string by including all common first male/female names of a certain language, say German. Then such query would (approximately) generate all publications on which at least one author with a German male/female first name is listed. Another consideration, however, is the limit for the number of search terms imposed by the WoS. A WoS advanced search query cannot contain more than 16,000 Boolean operators, and if a full list of male/female names of a certain language is to be repeated 26 times, then the list cannot contain a large number of names. In consideration of such limitation and also given the fact that for several languages that we sampled from there are several hundreds of male/female names, we adopted two different sampling methods. One is called here the “long list of first names, A-C surname initials” (hereafter, the A-C method) and the other is called “short list of first names, A-Z surname initials” (hereafter, the A-Z method). In the A-C method, for each given language, we composed a long (as comprehensive as possible) list of male first names and a similar list of female first names, but only considered the first three letter of the alphabet for the surname initials (so that the list of first names only has to be repeated 3 times). In the latter, however, we composed a list of 30 most popular names of each given language, one for females and one for males, but repeated that list 26 times to include every letter of the alphabet for the surname initials.

Popular first names given to people born in the late 1950’s to 1990’s were obtained as the sample for this study to ensure that it was representative of scholars active in academia from 2000–2020 (given the average age of an academic is around 40 years). Government registries and records (i.e., registry of Births, Deaths and Marriages) were used to obtain the names, however, for certain countries (i.e., Iran, India) this information was not publicly available. Instead, a minimum of two non-government databases were used to compile the list of top 30 first names, and it was essential criteria that they had separate lists for respective decades (1950’s-1990’s). In countries where a common language is spoken (for example UK/Australia/USA, Brazil/Portugal), the list of top 30 names was compiled using a combination of the most common names in those countries. Unisex names were excluded from the lists, and if a first name had alternative spellings (i.e., addition of an accent), the name and its alternatives were counted as one. To ensure further accuracy, two and three letter names were excluded, as they were often shared across multiple languages. For example, ‘Jan’ is a common European male name, however, in English it is a female name, and is also an abbreviation of the word January. All lists of names are accessible in the Online Supplementary Material of the paper.

In total, 14 different languages were considered for both A-C and A-Z methods. This includes (in alphabetical order) Arabic, Dutch, English, French, German, Hindi, Italian, Japanese, Korean, Persian, Portuguese, Russian, Spanish and Turkish. These languages were selected based on two criteria: (1) they are amongst the most widely spoken languages around the world, and (2) the languages allow for the female and male names to be easily distinguished. In order to increase specificity of the resultant data, for each method (A-C or A-Z) and each language, the search query was combined with the name of a country where that language is predominantly spoken (e.g., CU = Germany AND AU = (A* Albert OR B* Albert OR C* Albert …)) (where CU is the Field Tag for countries and AND is a Boolean operator). Only countries that are included in the list of top 100 in terms of the quantity of their scholarly publications were considered. This resulted in 37 different combinations of country and language. For each query (i.e., associated with the male/female A-C/A-Z list of each country-language combination), the meta data of publication count (for all document types) for the period of 2006–2020 was exported and stored for analysis. The reason for going back to only 2006 was that for some country-language combinations, the data before 2006 was scarce and/or lacking discernible patterns. Therefore, for the sake of consistency in comparisons, only the counts of publications from 2006 onwards were considered for all country-language combinations (i.e., a 15 year-long history of their publication records). It should also be noted that the resulting counts are based on number of publications with at least one female author or with at least one male author. Therefore, the counts do not take into account fractional contributions to papers and so cannot be inferred to directly reflect proportional contribution to publications.

Most previous studies on the gender gap in authorship are based on determination of the gender in individual documents of a sample of publications. Such an approach, while more nuanced than that of ours, often places a constraint on the number of documents that can be analysed. Typically, studies that use the abovementioned method rely on a sample of a several thousands of articles/pre-prints [43, 51]. Contrastingly, our method is query based, a novel approach in this research area. It is more suited for providing a bigger picture and broadening the scope and scale of the investigation, but that comes at a cost. The control over certain details such as position of authorship, or proportion of male versus female authors on each document is compromised. The series of formulated queries can be used to directly generate the set of publications in which male/female scientists are listed as authors. This directly examines the records of more than 75 scholarly publications without the pre-requisite of exporting their bibliographic data. It also does not rely on the use of any application programming interfaces. The method works only with the meta-data of publications and exempts the analyst from collecting a sample of publications. As such, it can be applied to the entire record of the WoS, without the need to export those records. The method can therefore be used as a benchmark for tracking overall productivity of male and female academics and their relative degree of representation in published documents. Our query-based approach also provides a foundation for replicating the data of overall gender productivity and observing changes in the trends over time. Additionally, it can be further modified to investigate author gender patterns within specific scholarly disciplines.

### Method for analysing the growth and gap

Consistent metrics were produced based on each of the abovementioned sets of meta data of publication count. Each metric is used to either quantify trends of productivity over the last 50 years (or 15 years, for the gender analyses), or to focus particularly on 2020 and the impact on the pandemic on such trends. For each dataset, the *Actual Growth* (AG) of publication counts were calculated associated with each year t, as Eq 1, where Pub_Cnt(t)_ signifies publication count in year t.


$$AG=(Pub_{Cnt(t)}-{Pub}_{Cnt(t-1)})/{Pub}_{Cnt(t-1)}*100$$ (1)

The averages of this quantity (AV-G) over the last five, 10 and 15 years (for genders) and five, 10, 20 and 50 years (for disciplines) are reported for each entity of analysis. This quantity is also specifically reported for the year 2020. From here on, when we mention AG without specifying a year, we refer to the 2020 version of this quantity. This is in contrast to the *Deviation of Growth* (DG) (in 2020) from the projected value. This quantity is calculated as below. Firstly, the data point of 2020 is excluded from the set of publication count. Then based on the historical data of 1970 (or, 2006)-2019 (depending on whether analysis of disciplines or genders is concerned), a polynomial curve of degree four is fitted to the data (this fitted curve has been visualised for each data set in the Results section). Using this regression analysis, we quantify the publications counts that was expected to manifest in 2020 (based on the record of 15 or 50 years of trend in publication count). This quantity is referred to as predicted counts of publications in 2020, pred_2020_ (as opposed to actual number of documents in 2020, act_2020_). The DG quantity is then calculated as Eq 2.


$$DG=({act}_{2020}-{pred}_{2020})/{act}_{2020}*100$$ (2)

The above are the metrics that are common between the disciplines and gender analyses. Additional quantities, however, we calculated in relation to gender analyses. For each set of data of country-language combination, the *Gap* (G) and *Relative Gap* (RG) between male and female publications as well as the *Ratio* (R) of female to male publications were calculated based on Eqs 3–5 (note that by male/female publications we mean publications on which at least one male/female author has been listed, at any authorship position). In this notation male_Cnt_ and female_Cnt_ respectively represent counts of male and female publications for the country-language combination of interest. Using the observation of ratios from 2006 to 2020, we also conducted an additional regression analysis on each set of data and predicted the year in which the female to male ratio of publication counts reaches .3, .5, .7 and .9 for each country-language combination. When no reasonable number could be achieved as a solution to the respective equation (e.g., more than 200 years in the future), then a dash sign “–” is reported in the respective tables of outcomes.


$$G=({male}_{Cnt}-{female}_{Cnt})$$ (3)

$$RG=({male}_{Cnt}-{female}_{Cnt})/{male}_{Cnt}*100$$ (4)

$$R={female}_{Cnt}/{male}_{Cnt}$$ (5)

## Results

### Overall research productivity trends

**Fig 1:**
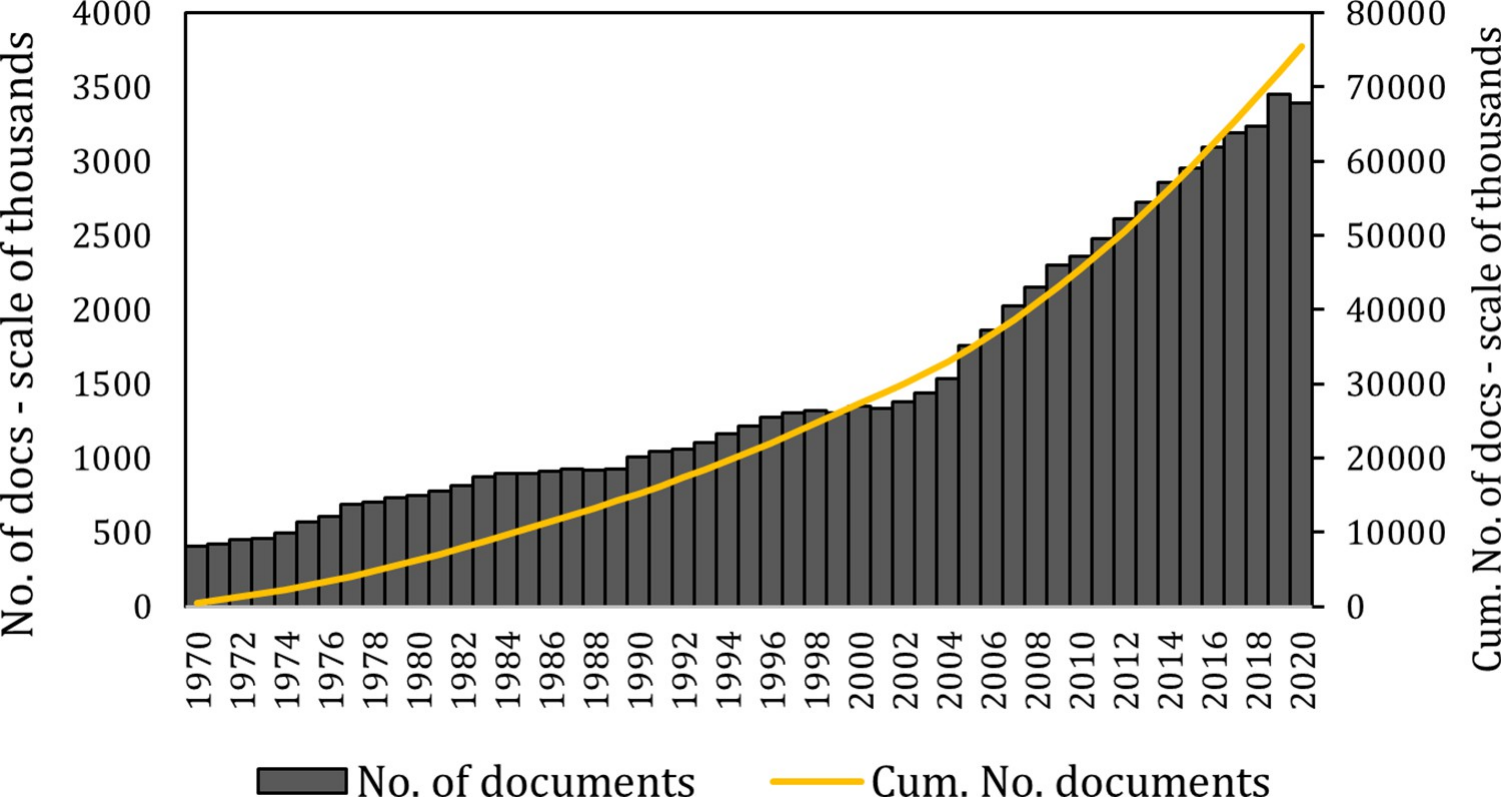
Cumulative growth of science during 1970–2020.

Fig 1 presents the number of documents and the cumulative number of documents per year, as recorded by Thompson Reuters Web of Science (hereafter, Web of Science [WoS]). During the current century, almost invariably the number of published documents grew compared to the previous year despite major challenges such as The Global Financial Crisis of 2007–2009. However, in 2020, the first year of Covid-19 pandemic, the number of publications declined from the previous year for the first time since 2002. This divergence from the established trend in 2020 (measured almost nine months into 2021, after stabilisation of 2020 publication count) prompted a revisiting of patterns in global research productivity. In this paper, the trends in productivity are revisited across different scientific disciplines (Jan 1970- Dec 2020) and author genders (Jan 2006- Dec 2020).

### Analyses across research fields

**Fig 2:**
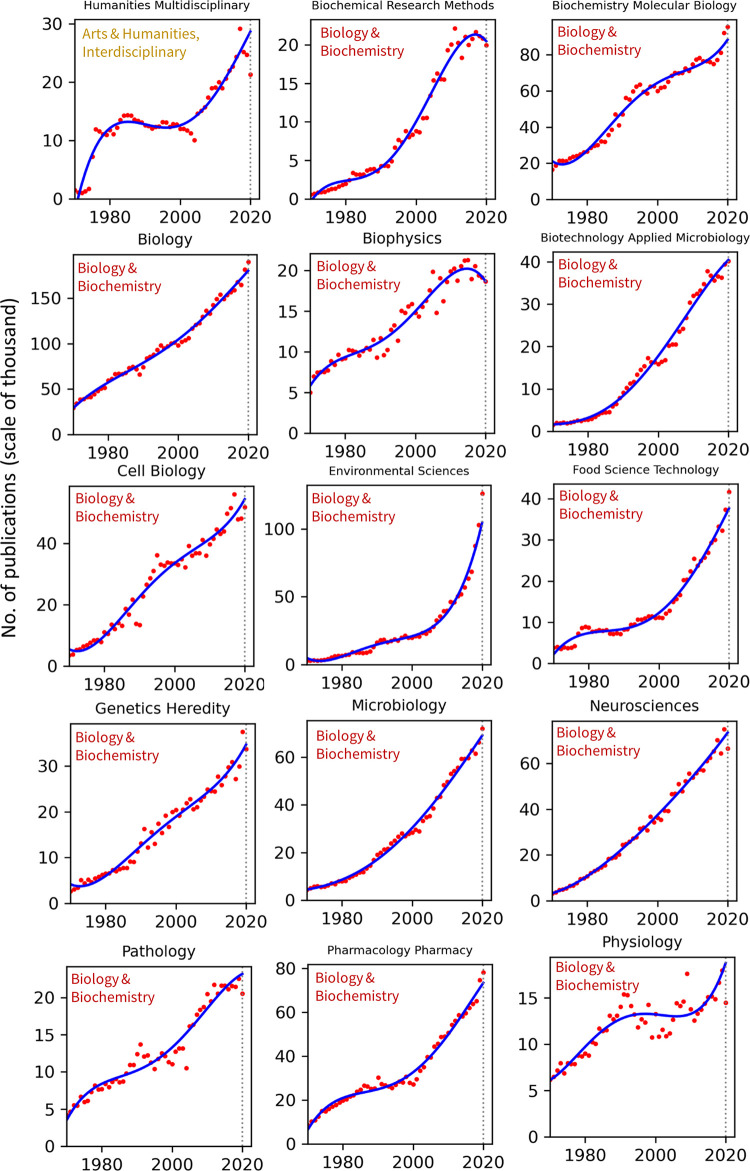
Temporal trends of research productivity in various scientific disciplines (part 1).

**Fig 3:**
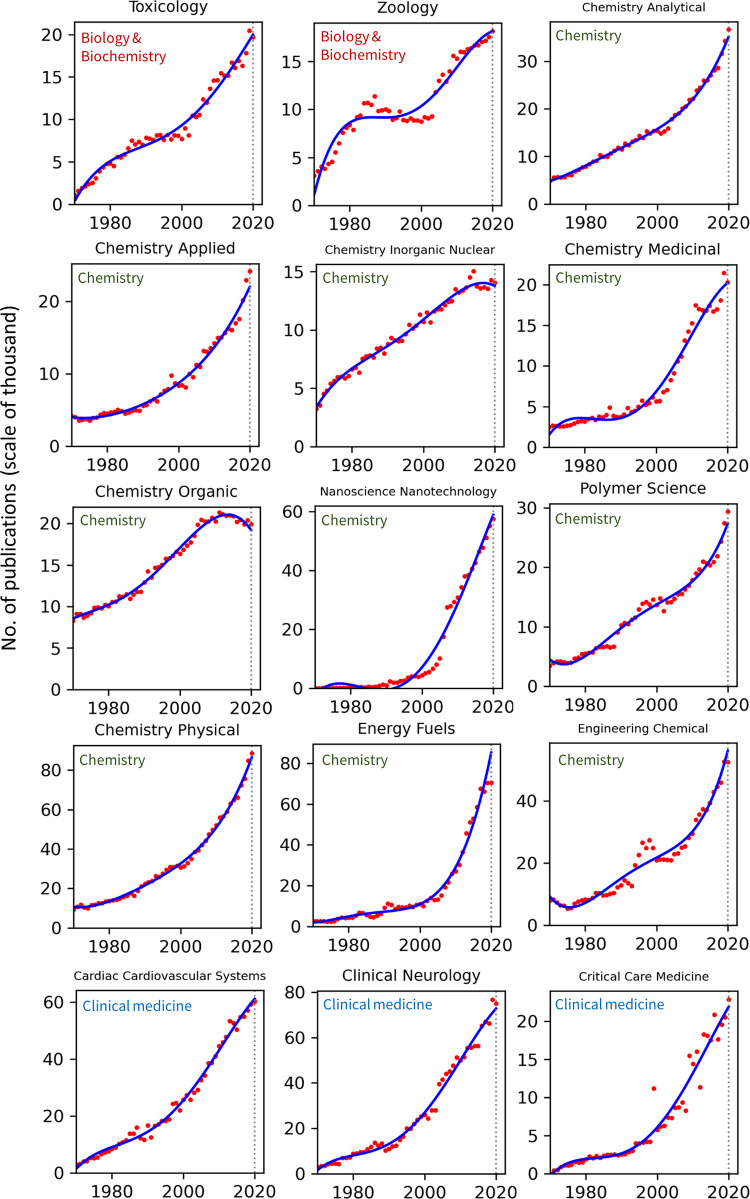
Temporal trends of research productivity in various scientific disciplines (part 2).

**Fig 4:**
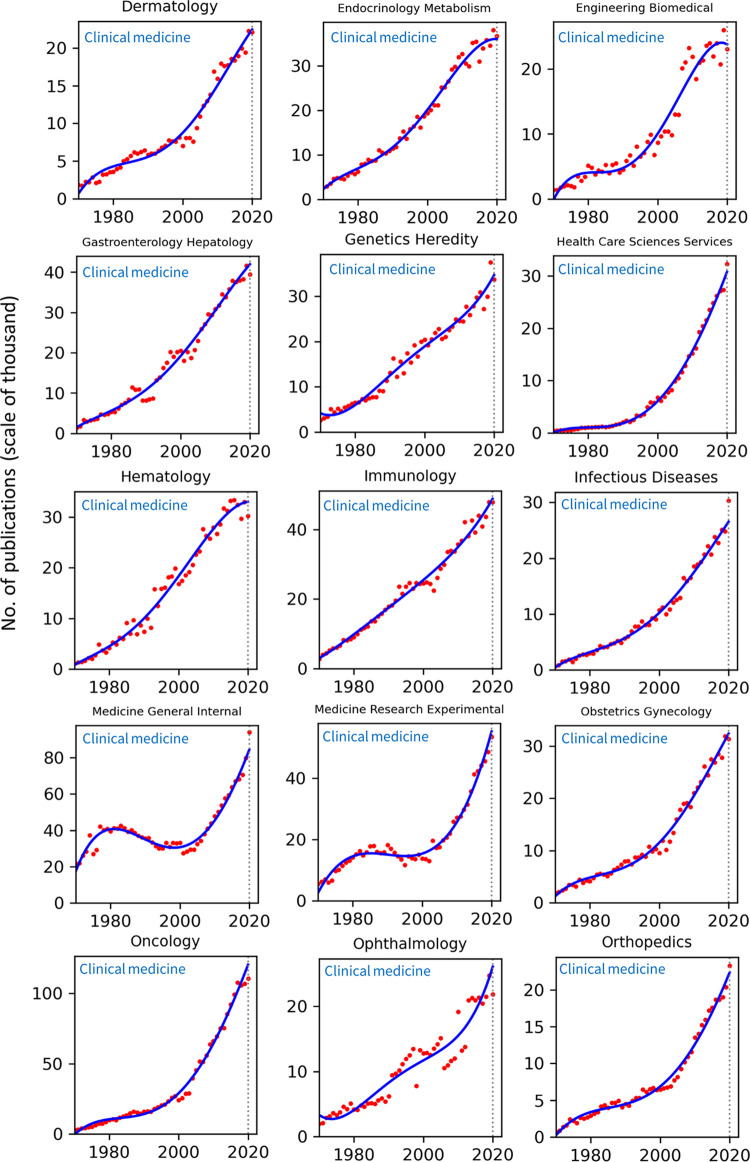
Temporal trends of research productivity in various scientific disciplines (part 3).

**Fig 5:**
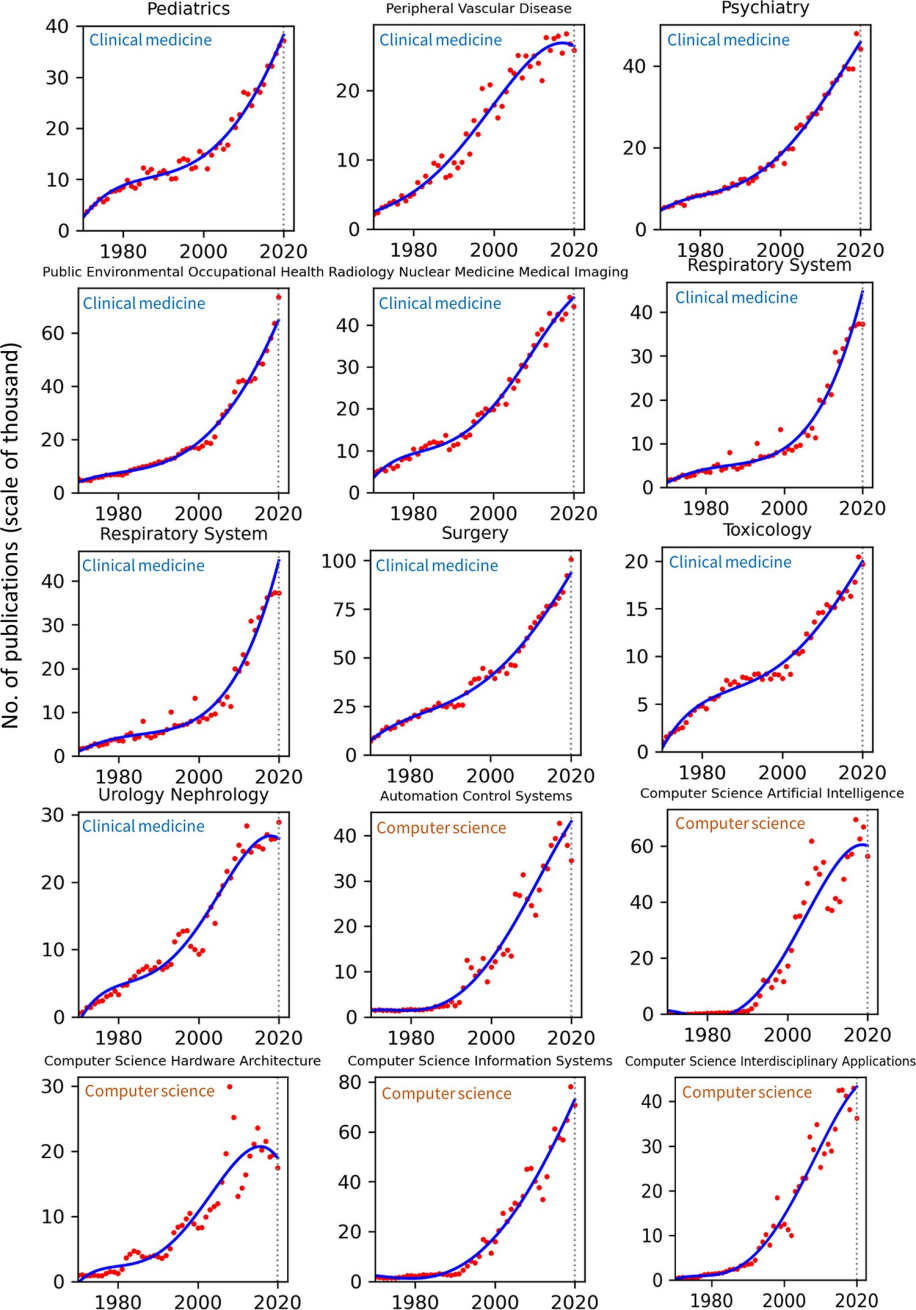
Temporal trends of research productivity in various scientific disciplines (part 4).

**Fig 6:**
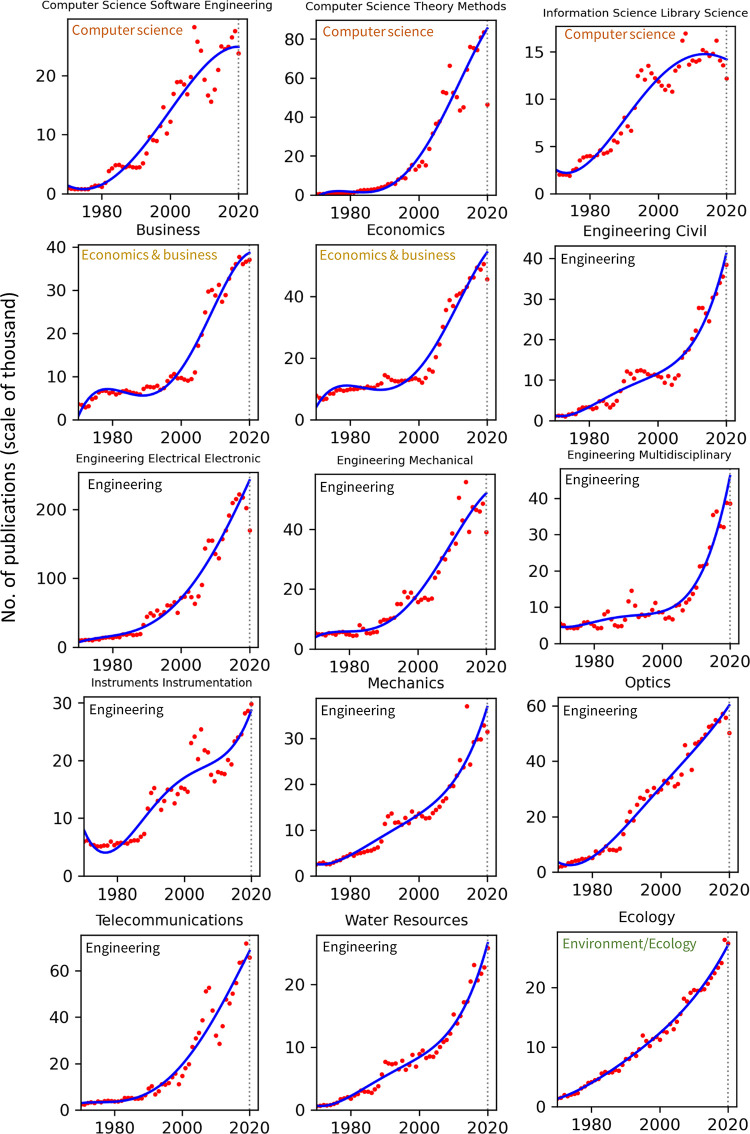
Temporal trends of research productivity in various scientific disciplines (part 5).

**Fig 7:**
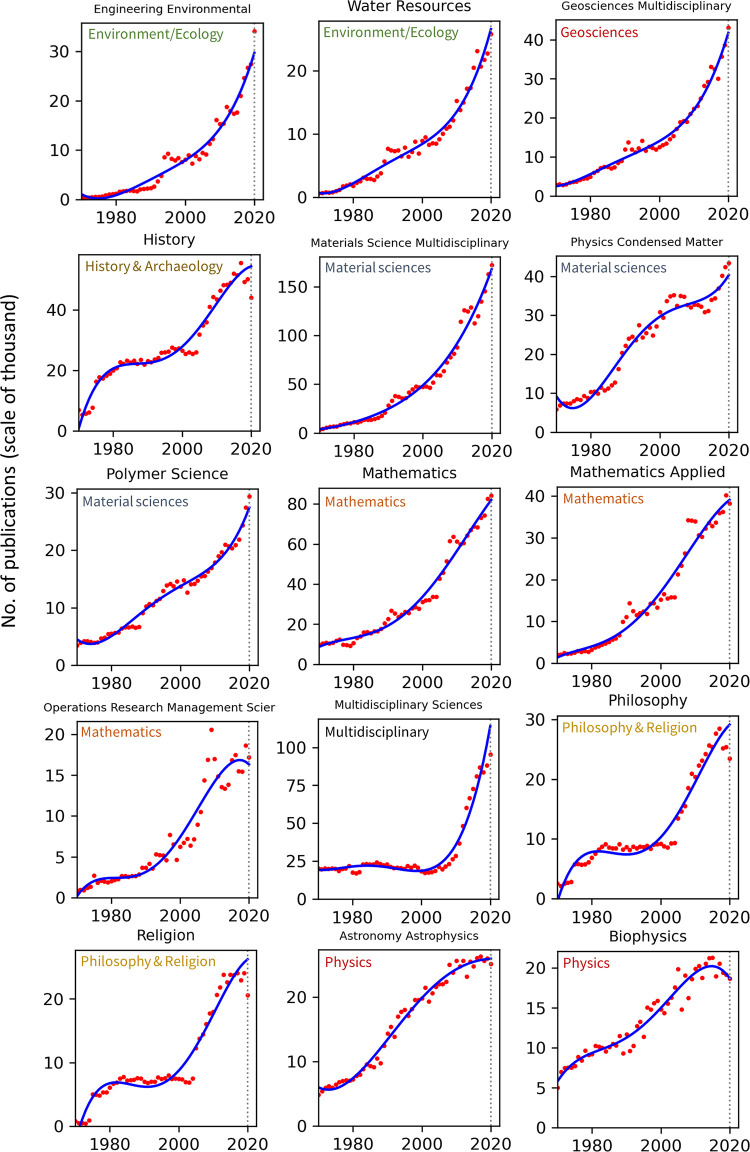
Temporal trends of research productivity in various scientific disciplines (part 6).

**Fig 8:**
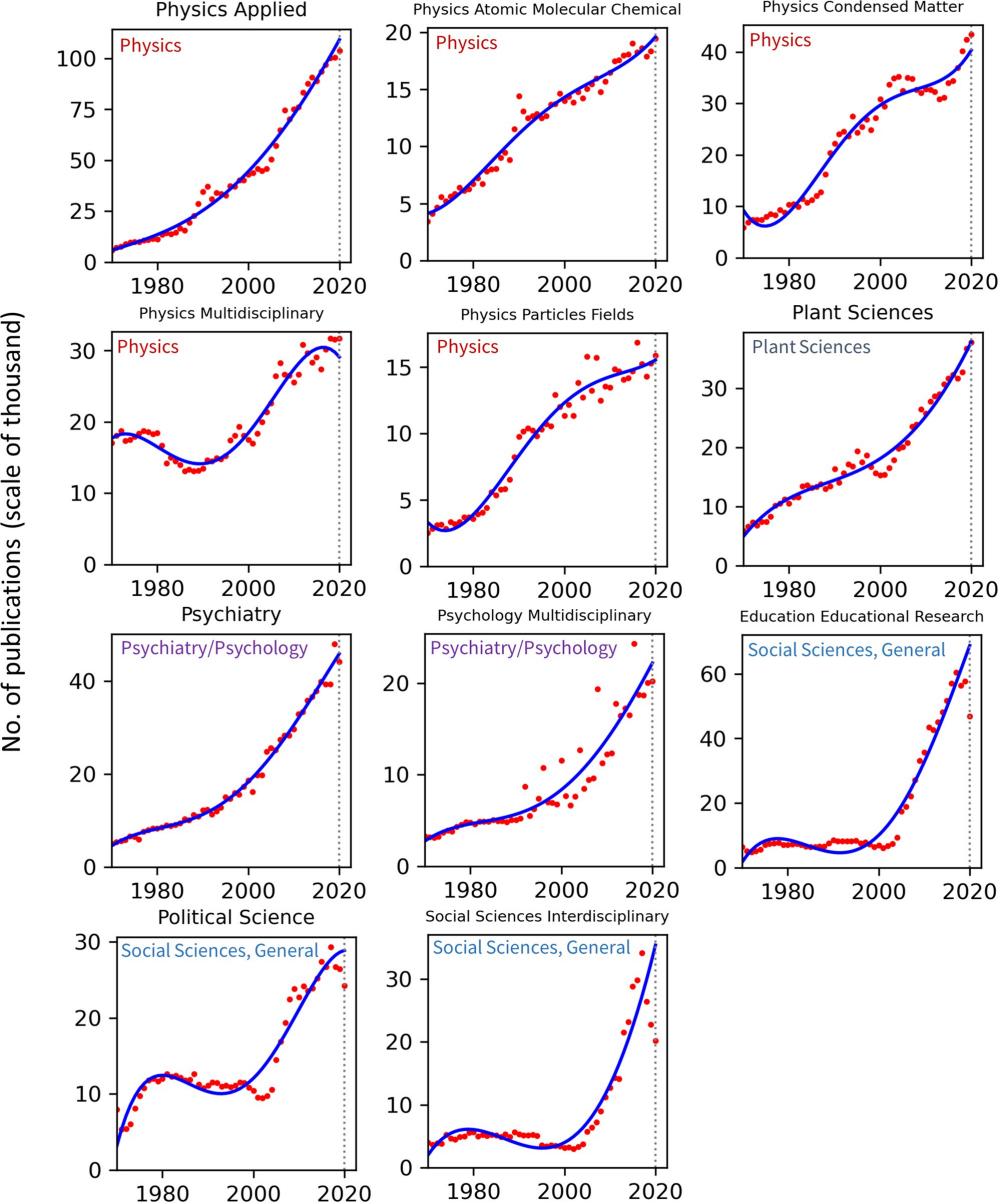
Temporal trends of research productivity in various scientific disciplines (part 7).

Publication counts across all top 100 WoS categories for the years 1970–2020 are presented in Figs 2–8. Categories that belong to the same broader area have been placed next to each other, while the name of the broad category that they identify with has been overlaid on each part of the figures. The blue trend curves are visualisations of polynomial curves fitted based on the data of 1970–2019. The prediction of the curves for the 2020 count of publication based on the trend is contrasted with the manifested number of publications for each category. The dashed vertical lines overlaid on each plot facilitates this comparison. The figures show the deviation of the actual number from the projected number of publications within each category. Table 1 provides various statistics related to WoS, including the actual and predicted number of publications in 2020, AG and DG, as well as average growth over the last 5, 10, 20 and 50 years. The cells containing statistics of actual and deviated growth in 2020 have been colour-coded to facilitate the comparison, with red indicating a decrease and green indicating an increase.

**Table 1:** Statistics of temporal trends of productivity in various domains of science.

Category	Actual 2020	Predicted 2020	AG% 2020	DG% 2020	AV-G 5y%	AV-G 10y%	AV-G 20y%	AV-G 50y%
**Arts & Humanities, Interdisciplinary**								
HUMANITIES MULTIDISCIPLINARY	21319	28680	-13.64	-34.53	-0.44	1.57	3.62	9.70
**Biology & Biochemistry**								
BIOCHEMICAL RESEARCH METHODS	19947	20462	-4.35	-2.58	-0.01	0.23	4.66	7.97
BIOCHEMISTRY MOLECULAR BIOLOGY	95562	88346	3.71	7.55	4.77	2.57	2.41	3.75
BIOLOGY	190038	180260	4.63	5.15	3.98	2.97	3.41	3.92
BIOPHYSICS	18698	18693	-2.50	0.02	-2.36	-0.45	1.83	3.43
BIOTECHNOLOGY APPLIED MICROBIOLOGY	40174	40448	1.86	-0.68	1.83	2.37	4.91	7.09
CELL BIOLOGY	51815	54351	7.75	-4.89	1.25	2.97	2.57	6.76
ENVIRONMENTAL SCIENCES	126108	104528	22.39	17.11	17.48	12.57	9.92	8.19
FOOD SCIENCE TECHNOLOGY	41683	37677	11.43	9.61	7.45	5.28	7.02	5.49
GENETICS HEREDITY	33733	34666	-10.03	-2.76	3.47	3.80	2.91	6.16
MICROBIOLOGY	71978	69121	8.64	3.97	4.05	3.84	4.86	5.91
NEUROSCIENCES	66554	73525	-11.19	-10.47	1.77	2.41	3.36	6.69
PATHOLOGY	20504	23178	-8.91	-13.04	-0.95	0.17	3.93	3.78
PHARMACOLOGY PHARMACY	78192	73340	4.83	6.21	5.66	4.51	5.53	4.74
PHYSIOLOGY	14464	18752	-19.38	-29.65	-0.19	0.89	1.08	2.30
TOXICOLOGY	19689	19968	-3.80	-1.42	4.38	3.19	5.18	6.47
ZOOLOGY	18164	18151	3.47	0.07	1.76	1.74	3.93	3.88
**Chemistry**								
CHEMISTRY PHYSICAL	88519	86533	4.49	2.24	6.45	5.53	5.47	4.75
CHEMISTRY APPLIED	24182	22020	5.35	8.94	8.62	5.55	5.73	3.85
CHEMISTRY INORGANIC NUCLEAR	14053	13767	-1.38	2.04	0.51	0.72	1.48	3.20
CHEMISTRY MEDICINAL	20318	20222	-5.38	0.47	3.46	3.18	6.92	4.68
CHEMISTRY ORGANIC	19917	19208	-2.52	3.56	-0.92	-0.16	1.02	1.83
CHEMISTRY PHYSICAL	88519	86533	4.49	2.24	6.45	5.53	5.47	4.75
ENERGY FUELS	70603	85248	0.47	-20.74	6.15	10.33	10.25	7.62
ENGINEERING CHEMICAL	52459	56119	-0.24	-6.98	6.04	6.08	4.81	4.43
NANOSCIENCE NANOTECHNOLOGY	57381	58831	4.27	-2.53	6.15	6.44	14.82	18.97
POLYMER SCIENCE	29409	27439	7.18	6.70	7.65	5.18	4.07	4.65
**Clinical Medicine**								
CARDIAC CARDIOVASCULAR SYSTEMS	60258	61206.73	1.74	-1.57	3.67	3.145	4.463	6.8078
CLINICAL NEUROLOGY	74851	72769.38	-2.5	2.78	6.188	4.313	5.855	7.3554
CRITICAL CARE MEDICINE	22846	22257.41	11.22	2.58	-7.048	1.207	6.8205	10.6254
DERMATOLOGY	22099	22446.55	-0.65	-1.57	3.952	3.462	6.259	5.7086
ENDOCRINOLOGY METABOLISM	36717	36005.65	-3.44	1.94	3.626	1.526	3.513	5.8438
ENGINEERING BIOMEDICAL	23048	23806.15	-11.32	-3.29	0.268	1.307	6.15	8.0528
GASTROENTEROLOGY HEPATOLOGY	39448	41967.99	-5.19	-6.39	0.932	2.561	3.5415	7.9586
GENETICS HEREDITY	33733	34665.56	-10.03	-2.76	3.466	3.804	2.9135	6.158
HEALTH CARE SCIENCES SERVICES	32263	30746.6	17.91	4.7	6.652	7.988	8.37	9.3542
HEMATOLOGY	30219	33055.71	-8.41	-9.39	-1.604	1.032	3.228	9.1196
IMMUNOLOGY	47858	48799.12	0.01	-1.97	4.34	3.317	3.6925	6.3486
INFECTIOUS DISEASES	30319	26535.15	22.01	12.48	8.404	5.376	5.7335	9.066
MEDICINE GENERAL INTERNAL	94026	84526.79	17.75	10.1	8.258	7.097	5.582	3.5112
MEDICINE RESEARCH EXPERIMENTAL	53573	55368.62	10.17	-3.35	5.346	7.1	7.6415	5.35
NUTRITION DIETETICS	23568	21393.85	8.45	9.23	7.622	7.444	7.532	7.0384
ONCOLOGY	110408	120383.12	3.31	-9.03	3.794	5.376	8.2	7.9152
OPHTHALMOLOGY	21851	26114.41	-11.7	-19.51	1.276	3.149	4.5725	6.8134
ORTHOPEDICS	23261	22342.5	14.16	3.95	5.908	5.656	6.7325	7.9124
PEDIATRICS	37197	38252.52	2.56	-2.84	5.478	3.42	5.2945	5.7514
PERIPHERAL VASCULAR DISEASE	25788	26405	-3.51	-2.39	-1.054	0.899	2.406	6.6178
PSYCHIATRY	44193	45902.39	-7.91	-3.87	3.614	4.318	4.8125	4.757
PUBLIC ENVIRONMENTAL OCCUPATIONAL HEALTH	73492	64840.34	15.35	11.77	8.682	5.988	7.8965	5.5964
RADIOLOGY NUCLEAR MEDICINE MEDICAL IMAGING	44460	46476.37	-4.74	-4.54	1.748	2.717	4.5365	5.0382
RESPIRATORY SYSTEM	37279	44691.24	-0.34	-19.88	3.326	7.651	9.715	9.0338
SURGERY	100636	93422.46	9.14	7.17	5.548	4.401	4.5295	5.8338
TOXICOLOGY	19689	19967.97	-3.8	-1.42	4.38	3.194	5.1835	6.4662
UROLOGY NEPHROLOGY	28926	26467.83	9.34	8.5	2.826	1.569	6.735	9.4898
**Computer Science**								
AUTOMATION CONTROL SYSTEMS	34536	43057.71	-8.77	-24.67	-1.58	4.071	8.0355	9.0812
COMPUTER SCIENCE ARTIFICIAL INTELLIGENCE	56305	60081.84	-15.71	-6.71	0.9	4.83	7.767	19.8746
COMPUTER SCIENCE HARDWARE ARCHITECTURE	17454	18952.39	-10.27	-8.58	-5.514	3.509	5.9985	8.077
COMPUTER SCIENCE INFORMATION SYSTEMS	70854	72944.52	-9.4	-2.95	3.628	6.855	8.827	9.2326
COMPUTER SCIENCE INTERDISCIPLINARY APPLICATIONS	36321	43295.23	-15.52	-19.2	-2.64	4.403	7.9415	11.9276
COMPUTER SCIENCE SOFTWARE ENGINEERING	23785	24902.11	-13.71	-4.7	-0.706	2.73	4.573	8.6768
INFORMATION SCIENCE LIBRARY SCIENCE	12171	14167.87	-10.36	-16.41	-3.174	-1.215	0.579	4.041
**Economics & Business**								
BUSINESS	37153	38676	1.35	-4.10	1.23	2.80	7.76	5.52
ECONOMICS	45657	54363.78	-9.86	-19.07	0.016	2.258	6.9985	3.9658
**Engineering**								
ENGINEERING CIVIL	38462	41131.86	8.15	-6.94	9.654	7.143	7.259	8.762
ENGINEERING ELECTRICAL ELECTRONIC	169879	242180	-15.96	-42.56	-3.85	2.80	5.52	7.04
ENGINEERING MULTIDISCIPLINARY	38588	46104.52	-0.58	-19.48	2.23	10.695	9.2205	6.2816
MECHANICS	31546	36892.23	-4.03	-16.95	5.64	7.091	5.46	6.056
**Environment/Ecology**								
ECOLOGY	27429	27107.22	-2.05	1.17	5.03	3.514	4.7325	6.558
ENGINEERING ENVIRONMENTAL	34099	29726.14	24.28	12.82	14.332	8.792	8.2	11.1178
WATER RESOURCES	25835	26606.22	13.46	-2.99	5.064	5.842	5.867	8.7278
**Geosciences**								
GEOSCIENCES MULTIDISCIPLINARY	43105	41771.72	11.37	3.09	5.846	7.062	6.5415	5.8538
**History & Archaeology**								
HISTORY	43993	54436.68	-12.36	-23.74	-3.016	0.323	2.8445	4.7394
**Material Sciences**								
MATERIALS SCIENCE MULTIDISCIPLINARY	172164	167687.83	5.66	2.6	8.942	7.443	7.005	8.5788
PHYSICS CONDENSED MATTER	43381	40334.45	2.38	7.02	5.032	2.922	1.867	4.3896
POLYMER SCIENCE	29409	27438.61	7.18	6.7	7.652	5.177	4.0655	4.65
**Mathematics**								
MATHEMATICS	84204	82028.56	1.89	2.58	4.196	3.286	5.3415	4.7424
MATHEMATICS APPLIED	38284	39111.6	-4.94	-2.16	3.242	1.386	5.209	6.6214
OPERATIONS RESEARCH MANAGEMENT SCIENCE	17153	16303.62	-7.96	4.95	0.99	0.742	6.2285	8.2826
**Multidisciplinary**								
MULTIDISCIPLINARY SCIENCES	95486	113999.43	8.34	-19.39	5.758	13.297	9.032	3.6022
**Philosophy & Religion**								
PHILOSOPHY	23503	29163.91	-7.66	-24.09	-1.262	1.633	5.3495	5.5128
RELIGION	20579	26226.77	-14.37	-27.44	-2.662	1.656	6.097	13.6982
**Physics**								
ASTRONOMY ASTROPHYSICS	25149	25920.55	-3.11	-3.07	0.362	-0.049	1.3755	3.6294
BIOPHYSICS	18698	18693.4	-2.5	0.02	-2.36	-0.449	1.8335	3.4324
PHYSICS APPLIED	103707	109130	3.30	-5.23	3.16	3.32	4.62	6.23
PHYSICS ATOMIC MOLECULAR CHEMICAL	19446	19589.66	6.05	-0.74	0.52	1.736	1.7355	3.8318
PHYSICS CONDENSED MATTER	43381	40334.45	2.38	7.02	5.032	2.922	1.867	4.3896
PHYSICS MULTIDISCIPLINARY	31692	29105.89	0.28	8.16	1.872	2.364	3.234	1.4366
PHYSICS PARTICLES FIELDS	15872	15527.29	3.78	2.17	1.954	1.924	2.3765	4.2846
**Plant & Animal Science**								
PLANT SCIENCES	37714	37796.12	2.86	-0.22	3.662	3.971	4.702	4.062
**Psychiatry/Psychology**								
PSYCHIATRY	44193	45902.39	-7.91	-3.87	3.614	4.318	4.8125	4.757
PSYCHOLOGY MULTIDISCIPLINARY	20184	22180.93	0.73	-9.89	6.442	7.019	7.63	6.844
**Social Sciences, General**								
EDUCATION EDUCATIONAL RESEARCH	46950	68752.09	-18.61	-46.44	-1.374	3.311	11.648	5.0056
POLITICAL SCIENCE	24250	28856.42	-8.16	-19	-2.174	0.855	4.838	2.8022
SOCIAL SCIENCES INTERDISCIPLINARY	20168	35393.32	-11.27	-75.49	-5.96	6.55	11.2985	4.4002

AG = Actual growth

DG = Deviation of growth

AV-G = Average growth

Green means sharp increase

Yellow means mild increase

Amber means mild decrease

Red means sharp decrease

5y means 5-year average, i.e., 2016–2020

10y means 10-year average, i.e., 2011–2020

15y means 15-year average, i.e., 2006–2020

According to WoS records, the biggest categories of contemporary research are Biology, Engineering Electrical Electronic, Biochemistry Molecular Biology, Chemistry Multidisciplinary, Medicine General Internal, and Material Science Multidisciplinary. The fastest growing areas over the past 5 years are Environmental Sciences and Engineering Environmental. The average annual growth of publication counts in these two categories over that period have respectively been 17.5% and 14.3%. Most categories have been steadily growing, but some categories fluctuate. This includes categories such as Arts & Humanities Multidisciplinary, Biology & Biochemistry, Medicine General Internal, Medicine Research Experimental, Physics Multidisciplinary and Political Science. Categories such as Philosophy, Religion and Education Educational Research have had sustained periods of negligible growth in the past but have been increasing in recent years. In addition to Environmental categories, categories that also show exponential patterns of growth are Chemistry Analytical, Nanoscience Nanotechnology, Energy Fuels, Health Care Sciences Services, Medicine Research Experimental (since about 2000), Respiratory System, Engineering Civil, Engineering Multidisciplinary, Mechanics, Water Resources, Geosciences Multidisciplinary, Material Sciences Multidisciplinary and Multidisciplinary Sciences (since 2000).

Within the various domains of science, this study uses the deviation from trends during 2020 as a proxy for the effect of the Covid-19 pandemic on research production. With this in mind, one can observe a mix of growth and decline in productivity across the broader areas. However, two areas appear to have been particularly affected are Computer Science and Social Sciences, General. In all categories of these two areas (within the list of top 100 categories), there have been fewer published papers in 2020 compared to 2019. Other categories, however, have grown markedly beyond expectation in 2020, i.e., their manifested number of publications in 2020 exceeded projected numbers. This includes categories such as Infectious Diseases, Environmental Sciences, Engineering Environmental, Medicine General Internal and Public Environmental Occupational Health.

### Analyses of author genders

**Fig 9:**
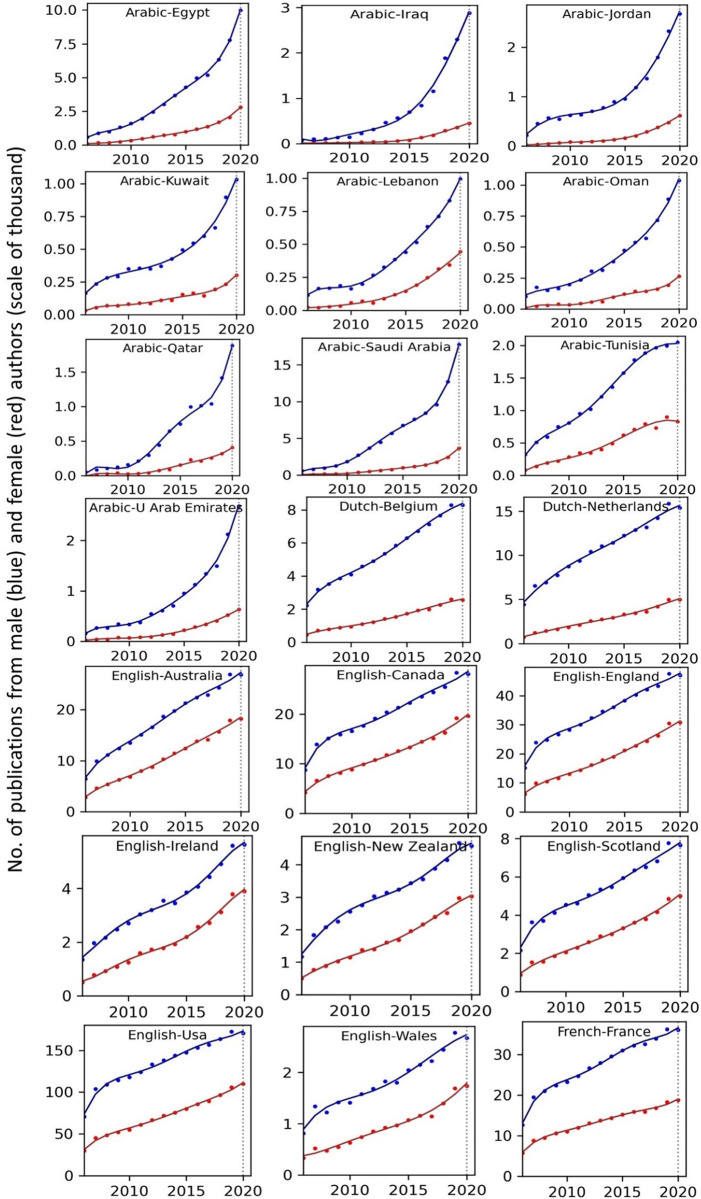
Temporal trends in male (blue lines) and female (red lines) total research productivity based on A-C method (part 1).

**Fig 10:**
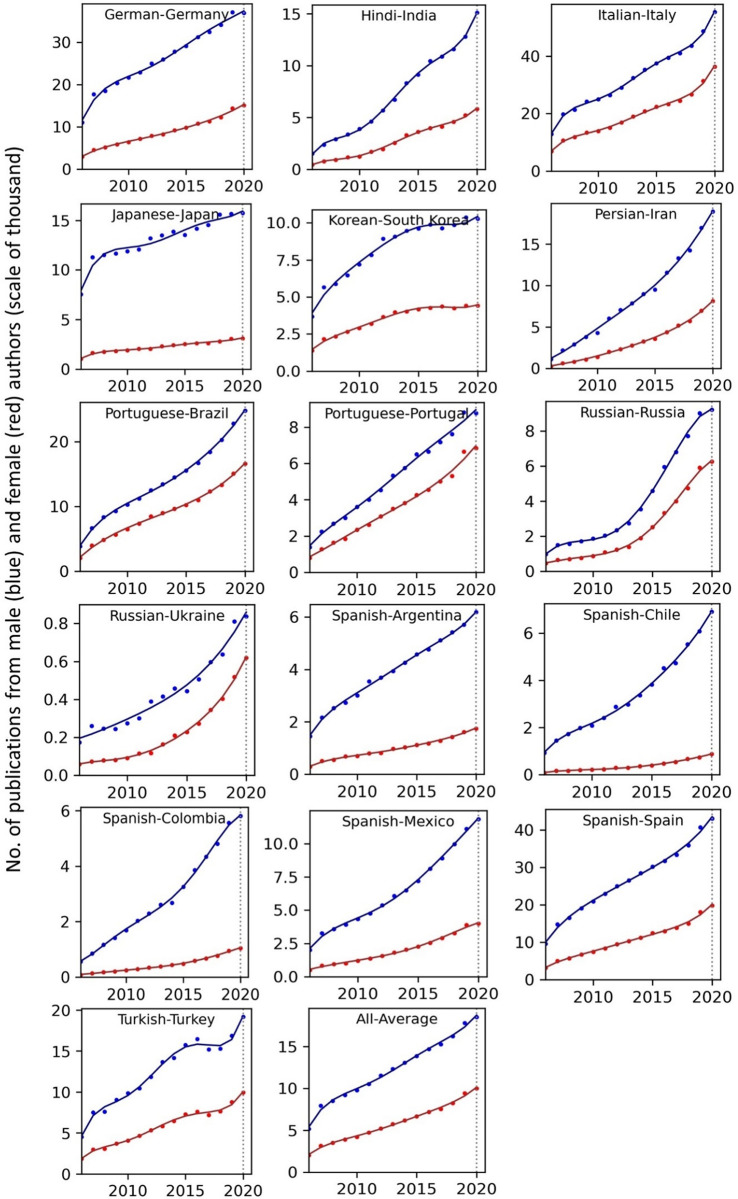
Temporal trends in male (blue lines) and female (red lines) total research productivity based on A-C method (part 2).

**Fig 11:**
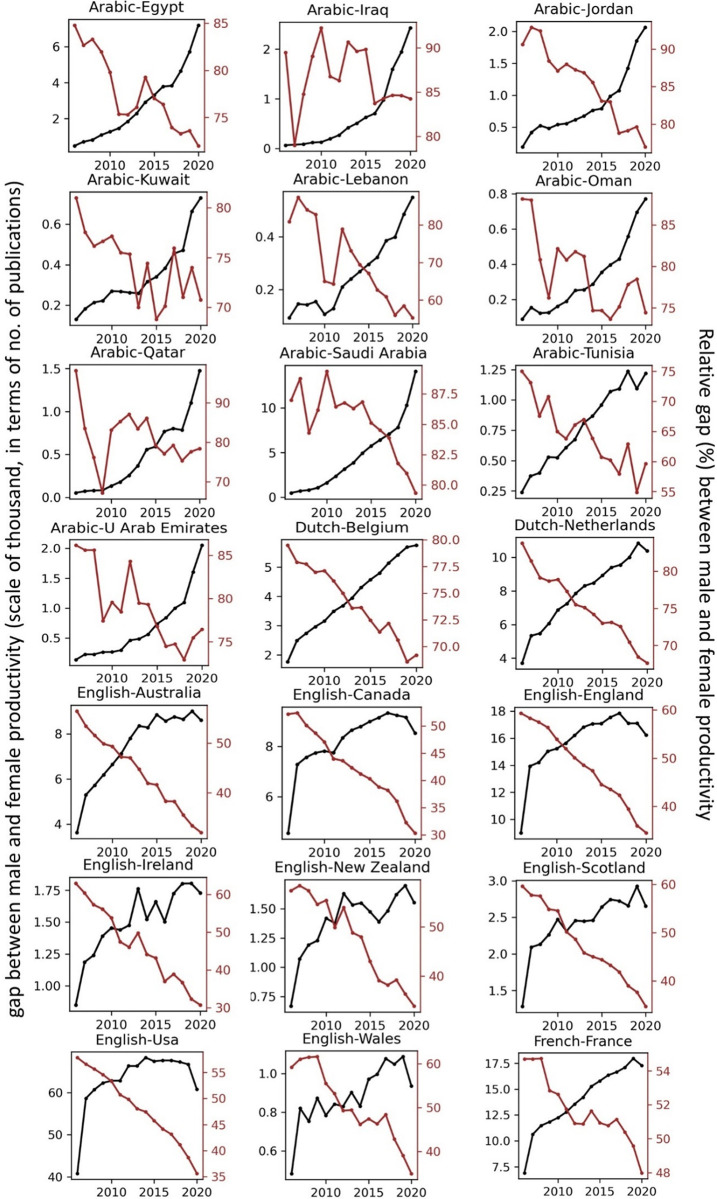
Gaps between male and female total research productivity, A-C method (part 1).

**Fig 12:**
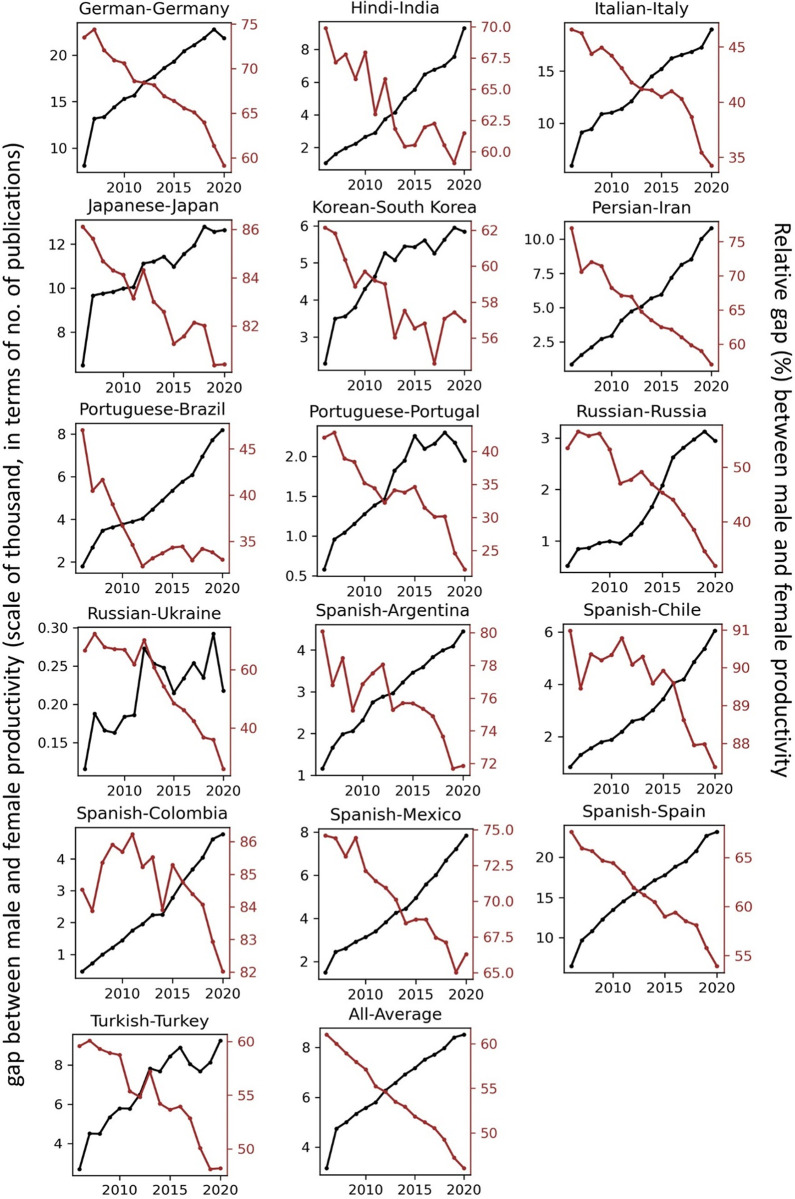
Gaps between male and female total research productivity, A-C method (part 2).

**Fig 13:**
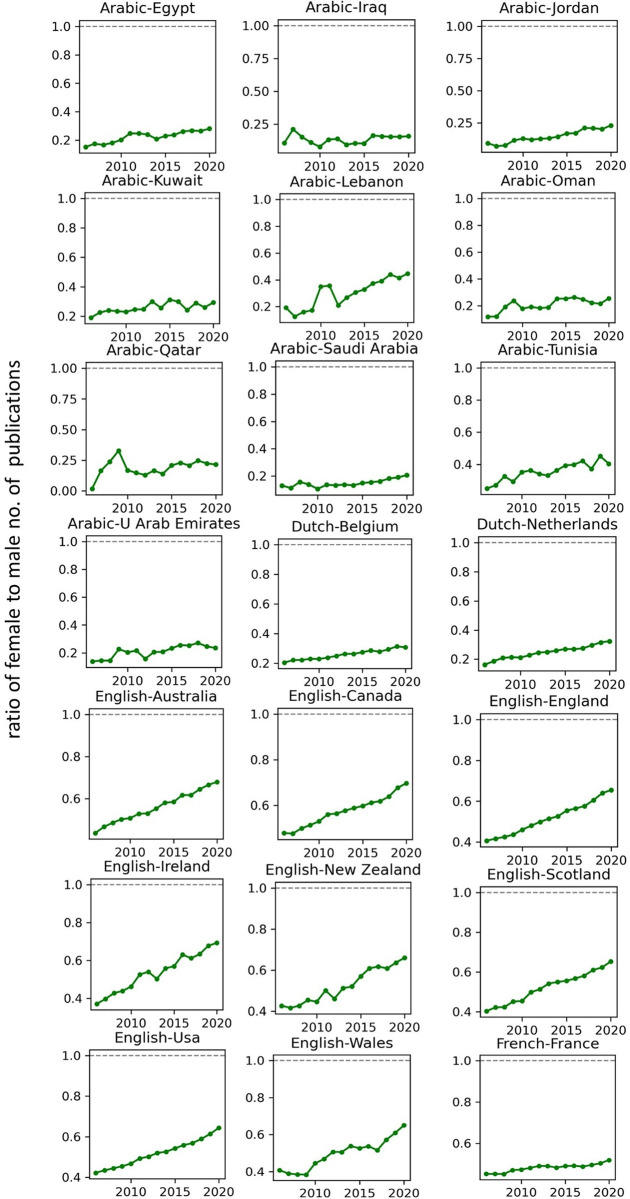
Ratio of female to male total research productivity, based on A-C method (part 1).

**Fig 14:**
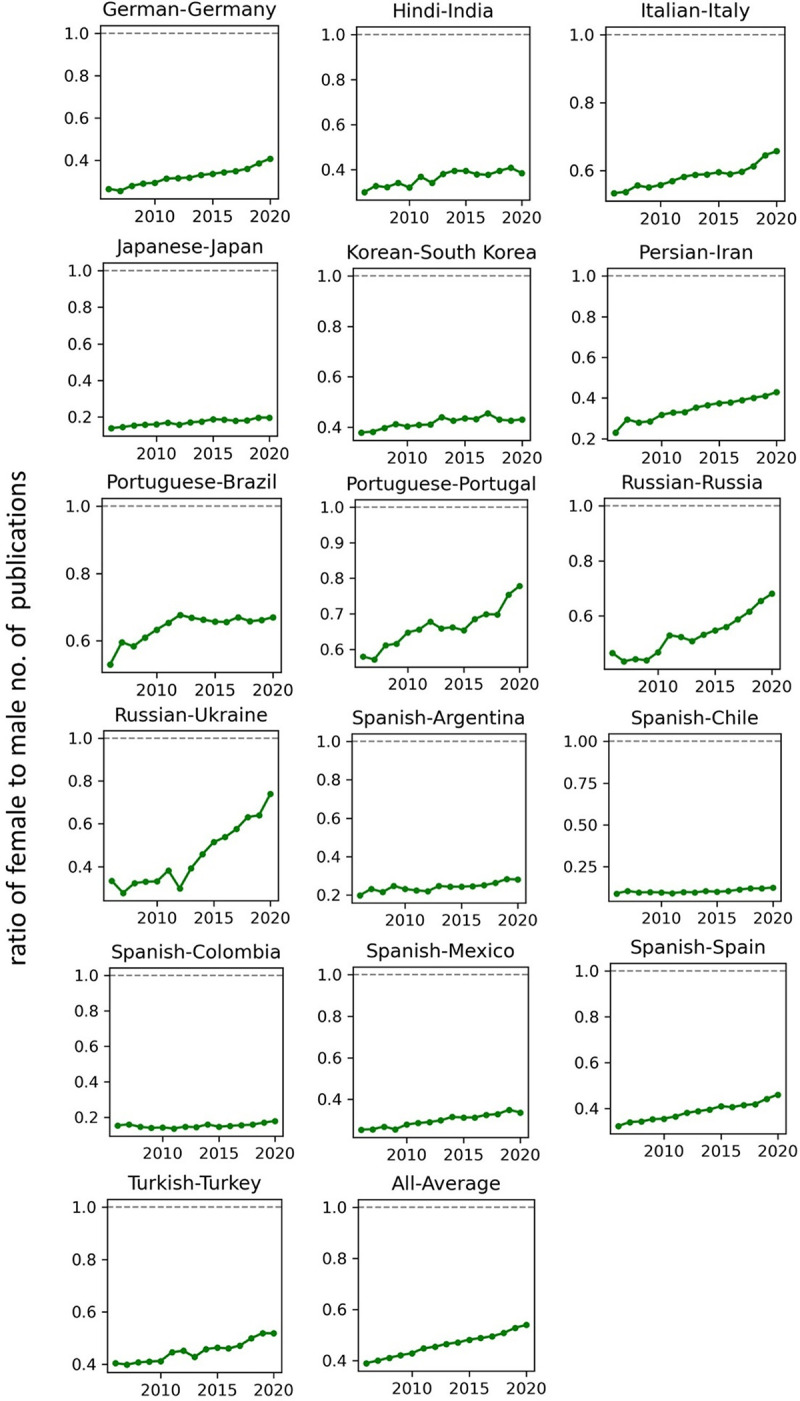
Ratio of female to male total research productivity, based on A-C method (part 2).

**Fig 15:**
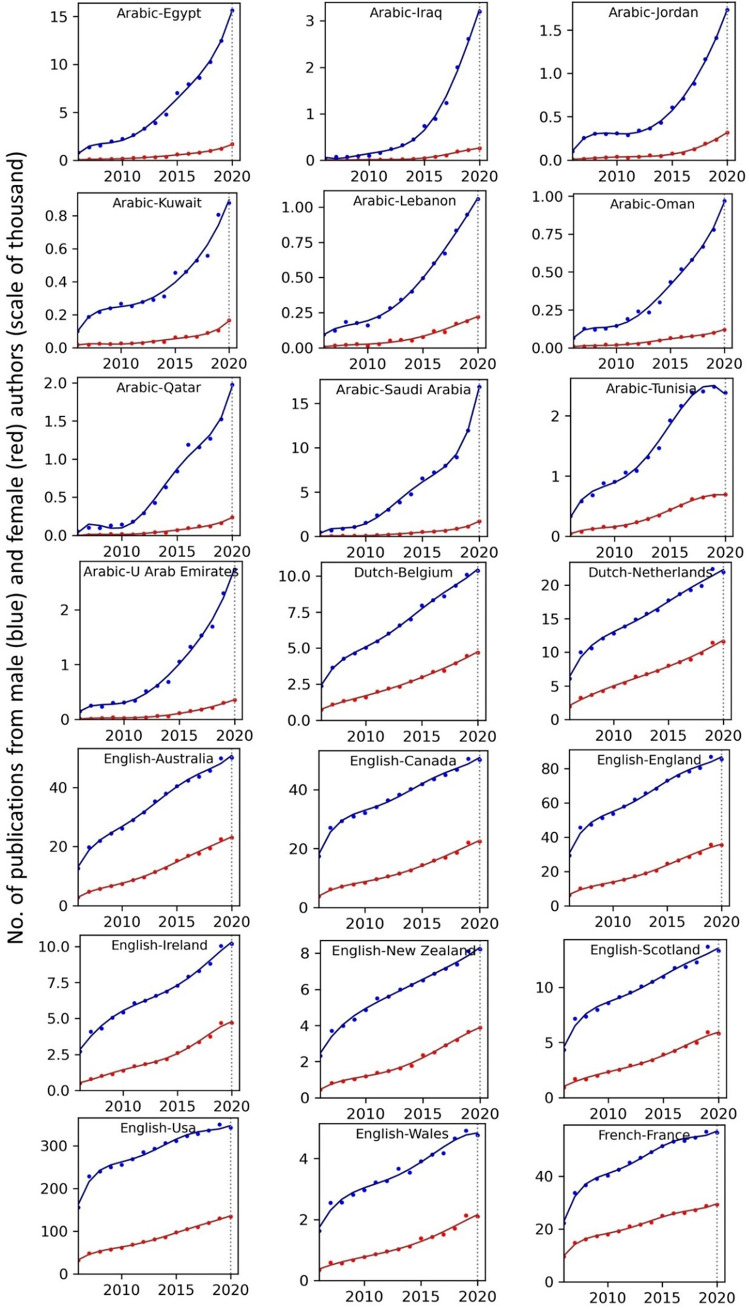
Temporal trends in male (blue lines) and female (red lines) total research productivity based on A-Z method (part 1).

**Fig 16:**
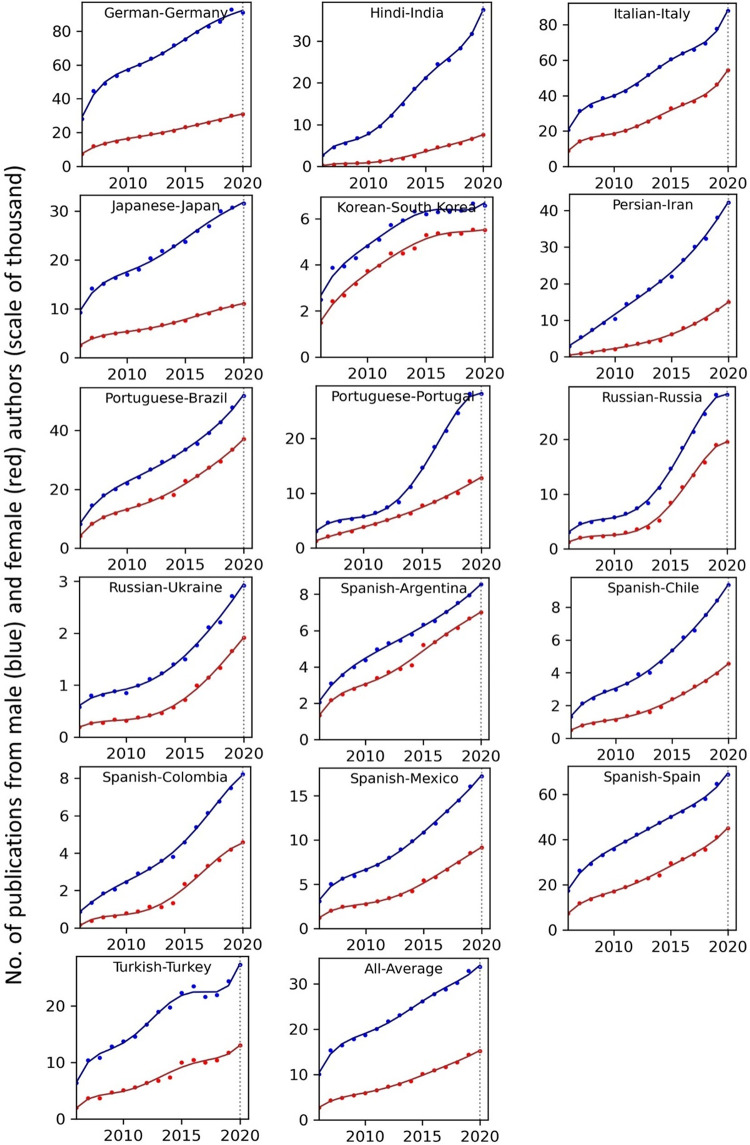
Temporal trends in male (blue lines) and female (red lines) total research productivity based on A-Z method (part 2).

**Fig 17:**
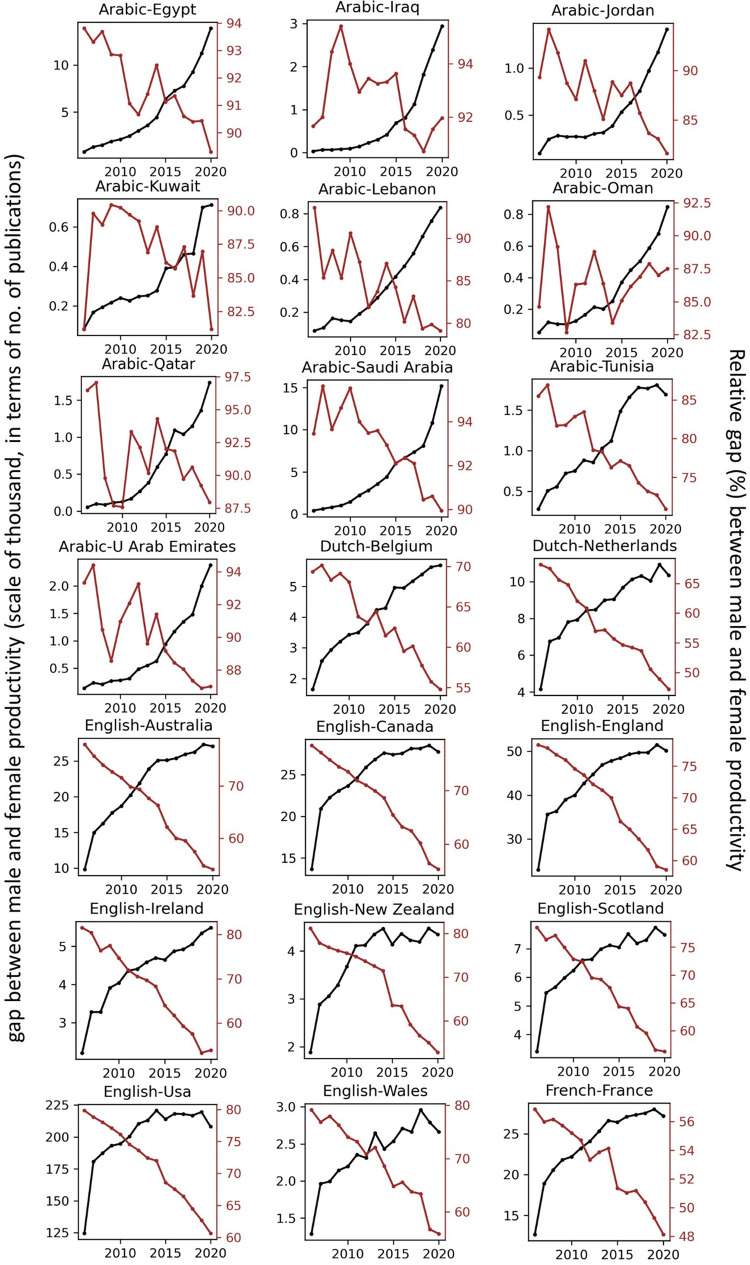
Gaps between male and female total research productivity, A-Z method (part 1).

**Fig 18:**
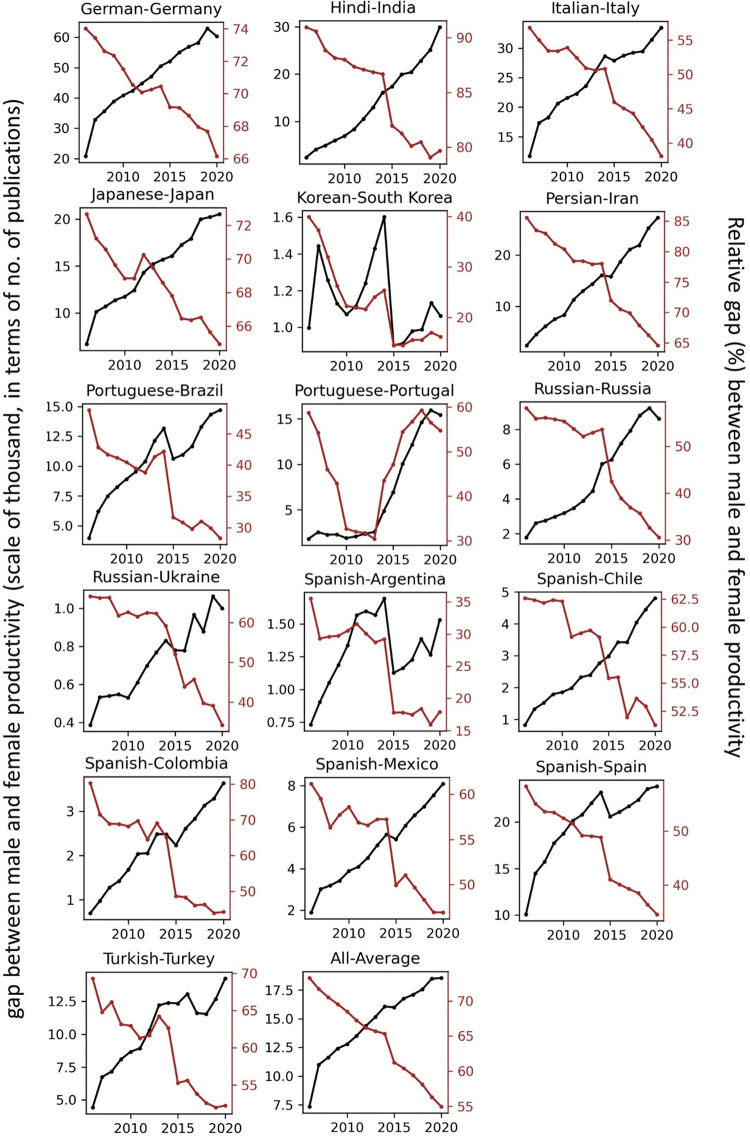
Gaps between male and female total research productivity, A-Z method (part 2).

**Fig 19:**
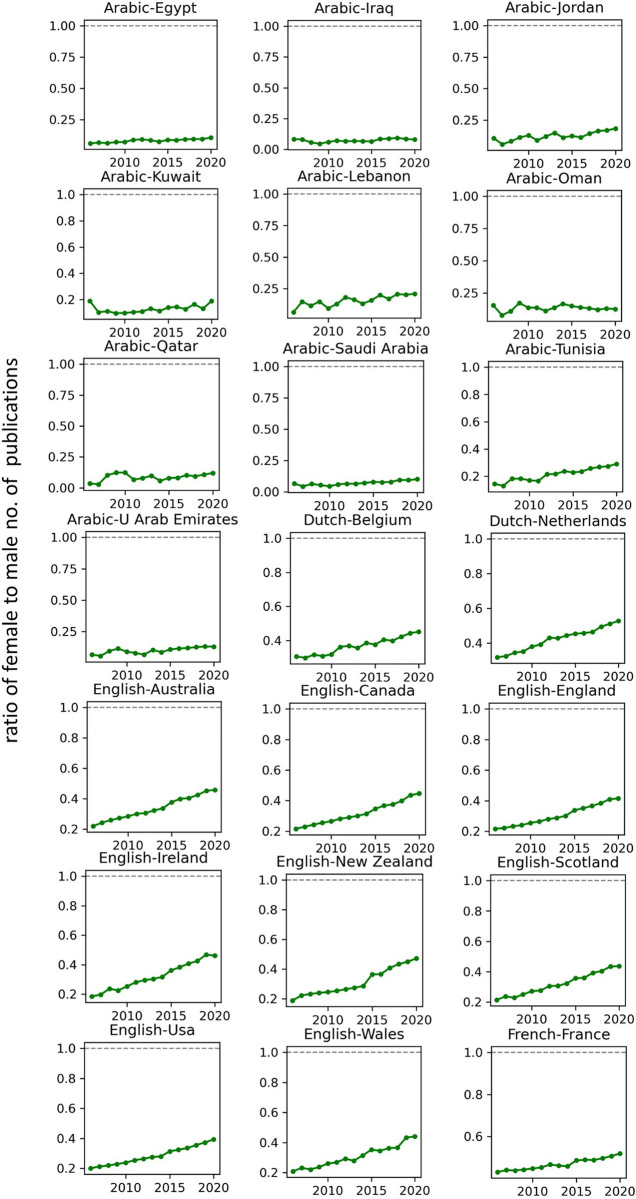
Ratio of female to male total research productivity, based on A-Z method (part 1).

**Fig 20:**
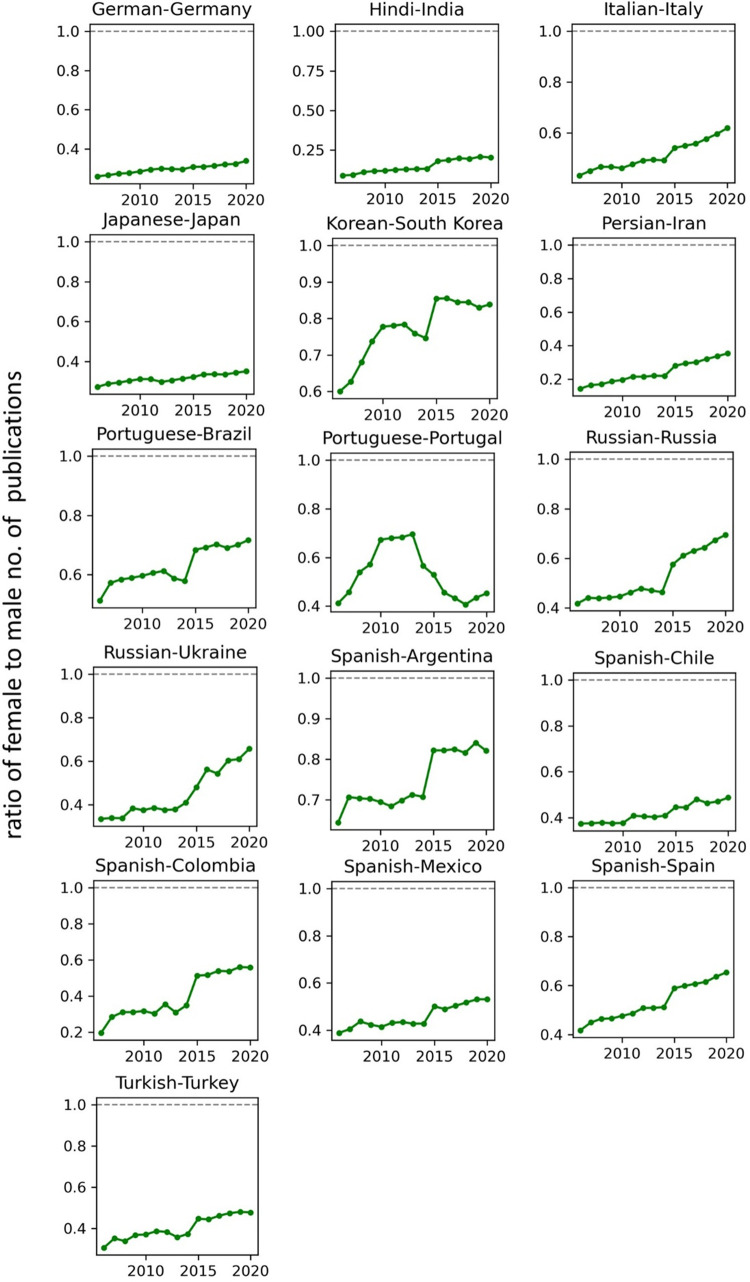
Ratio of female to male total research productivity, based on A-Z method (part 2).

The temporal record of publication counts of male- and female-authored publications (i.e., publications on which at least a male/female author has been listed) across language-country combinations have been visualised in Figs 9 and 10 for the period of 2006–2020. Overlaid on each plot are also the trend curves (fitted on the data of 2006–2019) along with a dashed vertical line at year = 2020 to facilitate evaluation of departures from forecast. The last unit of the plot set shows the average data across all country-language combinations. In Figs 11 and 12, the absolute gap (in number of documents) between male- and female-authored publications (left vertical axes, black curves) along with the relative gap (right vertical axes, red curves) have been visualised for each language-country combination. Similarly, the ratios of female to male publication counts across all language-country combinations have been visualised in Figs 13 and 14. The results presented in Figs 9–14 are all based on the A-C sampling method. The corresponding results from the A-Z sampling method have been presented in Figs 15–20.

Tables 2 and 3 summarise the results of the analyses based on the A-C and A-Z methods, respectively. In these tables, the actual and projected number of publications of male and females in 2020, along with the actual growth (AG) and deviation from the predicted growth (DG), have been reported, along with the average of the (actual) growth over the last 5, 10 and 15 years. The last four columns of the tables report the year in which the ratio of female to male publication counts of the language-country combination is expected to reach values of .3, .5, .7 and .9 based on the current trends. When no feasible solution could be found for the equation, a dash sign “–” has been symbolically reported. We also considered estimating number of years for reaching absolute parity i.e., r = 1.0. However, for many of the countries, no reasonable number can be found as a solution to report. While the ratio for all countries is “asymptotically approaching 1.0”, a solid parity (r = 1.0) cannot be achieved for any country that is showing a divergence pattern based on absolute numbers. That would require the absolute gap to close too, and most of the countries will not have that no matter how infinitesimally close their ratio gets to 1.0. The entities of Tables 2 and 3 in the AG and DG columns have been colour-coded to better demonstrate the positive (green) and negative (red) growth during 2020.

**Table 2:** Statistics of temporal trends of productivity of male and female scientists based on the A-C method.

Language-country	Actual 2020	Predicted 2020	AG% 2020	DG% 2020	AV-G 5y%	AV-G 10y%	AV-G 15y%	Year ratio = .3	Year ratio = .5	Year ratio = .7	Year ratio = .9
Arabic-Saudi Arabia_f	3666	3653	51.55	0.35	30.24	35.45	33.71				
Arabic-Saudi Arabia_m	17750	17747	39.69	0.02	21.89	26.06	28.42	2045	2085	2125	2166
Arabic-Egypt_f	2796	2779	36.26	0.61	23.43	24.61	29.03				
Arabic-Egypt_m	9984	10009	28.49	-0.25	18.72	20.31	22.97	2024	2048	2073	2097
Arabic-Iraq_f	453	453	28.33	0.08	47.02	50.87	43.89				
Arabic-Iraq_m	2879	2904	25.39	-0.88	33.62	35.92	30.71	2135	–	–	–
Arabic-Qatar_f	406	403	28.08	0.83	22.60	33.80	124.58				
Arabic-Qatar_m	1881	1881	32.75	0.02	21.37	29.01	29.88	2034	2069	2103	2138
Arabic-U Arab Emirates_f	632	631	21.31	0.14	23.36	25.63	30.00				
Arabic-U Arab Emirates_m	2682	2702	26.33	-0.74	23.33	23.78	23.62	2026	2050	2075	2100
Arabic-Kuwait_f	301	303	29.18	-0.52	15.61	15.42	19.34				
Arabic-Kuwait_m	1030	1042	14.96	-1.15	16.20	11.79	14.68	2022	2058	2094	2130
Arabic-Oman_f	265	262	38.74	1.08	17.83	23.57	26.64				
Arabic-Oman_m	1036	1049	16.80	-1.27	17.15	18.43	19.43	2026	2052	2078	2104
Arabic-Lebanon_f	444	436	28.70	1.76	25.34	24.42	26.06				
Arabic-Lebanon_m	994	996	19.61	-0.16	17.70	19.95	17.38	2014	2024	2033	2043
Arabic-Jordan_f	617	614	30.44	0.55	30.97	23.29	28.63				
Arabic-Jordan_m	2680	2723	15.32	-1.60	23.20	16.11	21.98	2029	2047	2065	2084
Dutch-Netherlands_f	4962	5096	-0.64	-2.71	8.77	10.61	15.69				
Dutch-Netherlands_m	15340	15640	-3.10	-1.95	4.77	5.90	9.77	2020	2039	2059	2079
Dutch-Belgium_f	2558	2613	-1.77	-2.16	8.28	10.71	13.68				
Dutch-Belgium_m	8303	8392	0.18	-1.07	5.71	7.37	10.22	2020	2047	2074	2101
English-USA_f	109757	111109	3.73	-1.23	6.58	7.17	10.26				
English-USA_m	170534	172961	-1.14	-1.42	2.99	3.79	6.98	2007	2013	2027	2040
English-England_f	30783	31313	1.22	-1.72	7.80	9.06	12.85				
English-England_m	47013	47710	-1.05	-1.48	4.23	5.27	9.09	2007	2014	2025	2036
English-Canada_f	19572	19954	1.97	-1.95	8.15	8.41	12.31				
English-Canada_m	28099	28607	-0.86	-1.81	4.82	5.45	9.42	2007	2009	2023	2036
English-Australia_f	18230	18541	1.74	-1.71	8.11	10.45	15.05				
English-Australia_m	26839	27297	-0.31	-1.70	4.83	7.22	11.31	2007	2011	2023	2035
English-Scotland_f	4995	5080	3.16	-1.69	8.68	9.33	14.35				
English-Scotland_m	7651	7787	-1.49	-1.78	5.28	5.46	10.45	2007	2013	2025	2036
English-Ireland_f	3902	3960	3.01	-1.48	12.47	12.34	16.42				
English-Ireland_m	5632	5712	0.72	-1.42	8.00	7.75	11.18	2007	2013	2022	2031
English-New Zealand_f	3026	3056	1.89	-1.00	9.28	10.44	14.37				
English-New Zealand_m	4581	4659	-1.86	-1.70	6.08	6.06	10.95	2007	2013	2024	2035
English-Wales_f	1738	1787	2.78	-2.83	10.48	10.96	13.43				
English-Wales_m	2673	2737	-3.78	-2.40	5.66	6.75	9.91	2007	2015	2026	2038
French-France_f	18745	18985	2.62	-1.28	4.31	5.50	9.46				
French-France_m	36037	36551	-0.52	-1.43	3.09	4.51	8.40	2007	2019	2068	2118
German-Germany_f	15071	15263	5.12	-1.27	9.08	9.06	12.97				
German-Germany_m	36912	37394	-0.57	-1.31	4.90	5.51	9.74	2012	2033	2055	2076
Hindi-India_f	5824	5889	11.40	-1.11	10.11	17.05	20.95				
Hindi-India_m	15122	15086	18.28	0.24	10.70	14.67	18.41	2007	2035	2065	2095
Italian-Italy_f	36377	36751	15.69	-1.03	10.38	10.16	13.20				
Italian-Italy_m	55342	55668	13.63	-0.59	8.15	8.34	11.55	2007	2007	2030	2056
Japanese-Japan_f	3080	3140	0.39	-1.95	4.07	5.09	8.67				
Japanese-Japan_m	15724	15923	0.54	-1.27	3.11	2.90	5.98	2049	2101	2154	–
Korean-South Korea_f	4416	4462	0.16	-1.05	1.18	4.42	9.30				
Korean-South Korea_m	10260	10430	-0.97	-1.65	1.37	3.71	8.29	2007	2035	2085	2136
Persian-Iran_f	8112	8133	16.82	-0.26	18.00	19.87	30.99				
Persian-Iran_m	18907	18975	11.56	-0.36	14.88	16.33	23.99	2011	2027	2044	2060
Portuguese-Portugal_f	6837	6977	2.84	-2.05	10.18	11.46	17.26				
Portuguese-Portugal_m	8785	8958	-0.40	-1.97	6.30	9.42	14.86	2007	2007	2018	2034
Portuguese-Brazil_f	16589	16681	10.04	-0.55	10.19	9.86	17.63				
Portuguese-Brazil_m	24771	24931	8.71	-0.64	9.74	9.21	15.16	2007	2007	2023	2050
Russian-Russia_f	6272	6321	6.14	-0.78	20.42	22.16	21.15				
Russian-Russia_m	9219	9262	2.05	-0.47	15.32	17.66	18.07	2007	2013	2025	2037
Russian-Ukraine_f	619	624	19.27	-0.83	22.19	21.60	18.92				
Russian-Ukraine_m	837	860	3.21	-2.71	13.88	12.18	12.70	2010	2016	2023	2029
Spanish-Spain_f	19785	20048	9.88	-1.33	9.96	10.37	14.78				
Spanish-Spain_m	42959	43443	5.53	-1.13	7.37	7.51	11.82	2007	2027	2050	2073
Spanish-Mexico_f	3985	4052	2.68	-1.67	12.23	12.74	16.51				
Spanish-Mexico_m	11828	11912	6.56	-0.71	10.47	10.57	14.16	2015	2044	2074	2103
Spanish-Colombia_f	1046	1056	9.99	-0.94	17.04	15.91	20.07				
Spanish-Colombia_m	5819	5842	4.43	-0.39	12.42	13.36	18.86	2113	–	–	–
Spanish-Argentina_f	1742	1760	7.80	-1.05	9.41	9.70	14.74				
Spanish-Argentina_m	6189	6191	8.41	-0.03	6.24	7.53	11.39	2028	2073	2118	2163
Spanish-Chile_f	873	870	19.26	0.31	17.90	16.08	19.43				
Spanish-Chile_m	6919	6915	13.56	0.05	12.71	12.89	15.96	2111	–	–	–
Turkish-Turkey_f	9923	10032	13.29	-1.10	6.62	9.52	13.69				
Turkish-Turkey_m	19154	19297	13.43	-0.75	4.29	7.10	11.86	2007	2020	2044	2067
All-Average_f	9979	10106	6.16	-1.27	8.47	9.10	12.67				
All-Average_m	18497	18708	3.89	-1.14	6.01	6.61	10.07	2007	2018	2038	2057

AG = Actual growth

DG = Deviation of growth

AV-G = Average growth

Green means sharp increase

Yellow means mild increase

Amber means mild decrease

Red means sharp decrease

5y means 5-year average, i.e., 2016–2020

10y means 10-year average, i.e., 2011–2020

15y means 15-year average, i.e., 2006–2020

**Table 3:** Statistics of temporal trends of productivity of male and female scientists based on the A-Z method.

Language-country	Actual 2020	Predicted 2020	AG% 2020	DG% 2020	AV-G 5y%	AV-G 10y%	AV-G 15y%	Year ratio = .3	Year ratio = .5	Year ratio = .7	Year ratio = .9
Arabic-Saudi Arabia_f	1695	1687	51.07	0.50	27.69	40.25	36.65				
Arabic-Saudi Arabia_m	16857	16800	41.23	0.34	21.48	27.95	30.06	2082	2141	2200	–
Arabic-Egypt_f	1669	1649	39.90	1.21	22.23	27.82	30.59				
Arabic-Egypt_m	15613	15578	25.18	0.22	17.56	21.85	24.57	2094	2167	–	–
Arabic-Iraq_f	257	259	16.29	-0.73	42.24	47.05	41.94				
Arabic-Iraq_m	3199	3238	22.33	-1.21	34.94	42.26	40.19	2167	–	–	–
Arabic-Qatar_f	238	234	45.12	1.82	30.08	36.32	51.36				
Arabic-Qatar_m	1975	1955	29.59	1.01	19.65	31.18	30.79	2087	2155	–	–
Arabic-U Arab Emirates_f	355	357	17.94	-0.57	25.87	32.37	32.85				
Arabic- U Arab Emirates_m	2732	2747	18.83	-0.55	21.32	25.23	24.65	2061	2106	2152	2198
Arabic-Kuwait_f	165	162	57.14	1.77	22.92	22.88	18.85				
Arabic-Kuwait_m	877	894	8.94	-1.99	15.00	13.75	18.55	2077	2149	–	–
Arabic-Oman_f	121	121	19.80	0.20	13.48	20.60	21.15				
Arabic-Oman_m	968	960	24.42	0.79	17.37	21.43	23.27	–	–	–	–
Arabic-Lebanon_f	221	222	15.71	-0.43	25.30	34.80	39.01				
Arabic-Lebanon_m	1058	1064	11.72	-0.60	16.52	21.02	19.81	2033	2058	2083	2109
Arabic-Jordan_f	318	318	33.61	0.10	34.48	27.49	30.82				
Arabic-Jordan_m	1733	1736	22.91	-0.16	23.40	19.43	26.01	2043	2075	2107	2139
Dutch-Netherlands_f	11572	11788	1.21	-1.87	7.68	9.19	14.46				
Dutch-Netherlands_m	21929	22286	-1.98	-1.63	4.44	5.61	10.42	2007	2020	2033	2047
Dutch-Belgium_f	4692	4748	4.90	-1.19	9.51	11.47	14.74				
Dutch-Belgium_m	10378	10473	2.72	-0.92	5.48	7.55	11.64	2008	2027	2045	2063
English-USA_f	134891	136055	3.28	-0.86	6.63	8.28	11.50				
English-USA_m	342983	347646	-2.03	-1.36	1.94	3.00	6.29	2016	2030	2045	2060
English-England_f	35485	35995	-0.40	-1.44	7.64	10.16	13.72				
English-England_m	85627	86889	-1.64	-1.47	3.25	4.83	8.59	2014	2027	2041	2054
English-Canada_f	22402	22773	1.63	-1.66	9.24	10.26	14.36				
English-Canada_m	50169	50928	-0.78	-1.51	3.69	4.57	8.50	2013	2026	2038	2050
English-Australia_f	22986	23307	1.80	-1.40	8.59	12.09	17.15				
English-Australia_m	50069	50807	0.31	-1.47	4.41	6.77	10.95	2012	2024	2036	2047
English-Scotland_f	5815	5932	-2.19	-2.01	8.49	9.75	15.26				
English-Scotland_m	13310	13558	-2.77	-1.86	4.07	4.57	9.23	2013	2025	2037	2050
English-Ireland_f	4697	4778	0.26	-1.73	12.68	13.36	18.03				
English-Ireland_m	10182	10298	1.55	-1.14	7.05	6.58	10.44	2014	2023	2033	2043
English-New Zealand_f	3876	3888	6.28	-0.32	10.43	12.77	18.20				
English-New Zealand_m	8223	8316	1.29	-1.12	4.84	5.45	10.18	2014	2024	2034	2044
English-Wales_f	2101	2157	-1.32	-2.65	9.15	10.81	15.09				
English-Wales_m	4764	4838	-3.15	-1.55	4.12	5.00	8.74	2014	2026	2039	2051
French-France_f	29327	29589	1.61	-0.89	3.23	5.04	8.94				
French-France_m	56541	57131	-0.65	-1.04	1.90	3.48	7.48	2007	2020	2053	2087
German-Germany_f	30881	31111	2.78	-0.75	5.90	6.65	11.57				
German-Germany_m	91244	92364	-1.86	-1.23	3.97	4.83	9.48	2015	2056	2097	2137
Hindi-India_f	7622	7593	14.72	0.38	14.88	23.77	28.91				
Hindi-India_m	37514	37508	18.09	0.02	12.19	17.06	21.36	2031	2053	2075	2097
Italian-Italy_f	54343	54503	17.38	-0.29	10.73	11.49	14.41				
Italian-Italy_m	87837	88320	12.94	-0.55	7.74	8.22	11.43	2007	2014	2030	2046
Japanese-Japan_f	11085	11113	4.79	-0.26	7.82	7.67	11.89				
Japanese-Japan_m	31621	31854	2.67	-0.74	5.99	6.43	9.80	2011	2054	2096	2139
Korean-South Korea_f	5502	5519	-0.49	-0.31	0.78	4.05	10.64				
Korean-South Korea_m	6563	6684	-1.49	-1.84	1.17	3.25	7.88	2007	2007	2010	2023
Persian-Iran_f	14961	14949	16.45	0.08	19.50	22.66	31.49				
Persian-Iran_m	42269	42421	10.87	-0.36	14.06	15.40	22.77	2018	2032	2046	2059
Portuguese-Portugal_f	12755	12938	3.99	-1.43	10.57	12.82	18.76				
Portuguese-Portugal_m	28189	28329	-0.07	-0.50	14.21	17.61	17.83	2007	2007	–	–
Portuguese-Brazil_f	37134	37102	10.87	0.09	10.16	11.10	18.53				
Portuguese-Brazil_m	51828	52212	8.38	-0.74	9.12	8.93	15.17	2007	2007	2020	2036
Russian-Russia_f	19575	19613	3.05	-0.19	18.68	23.43	22.67				
Russian-Russia_m	28189	28329	-0.07	-0.50	14.21	17.61	17.83	2007	2013	2023	2033
Russian-Ukraine_f	1917	1916	15.62	0.07	21.93	19.97	18.37				
Russian-Ukraine_m	2917	2949	7.20	-1.09	14.46	13.31	12.63	2008	2017	2025	2033
Spanish-Spain_f	45013	45244	9.25	-0.51	8.82	10.31	14.69				
Spanish-Spain_m	68831	69436	6.28	-0.88	6.57	6.78	10.87	2007	2013	2025	2037
Spanish-Mexico_f	9146	9198	7.26	-0.57	11.01	12.92	16.52				
Spanish-Mexico_m	17234	17345	7.25	-0.65	9.69	10.03	13.73	2007	2019	2038	2058
Spanish-Colombia_f	4589	4545	9.24	0.96	14.33	20.78	29.64				
Spanish-Colombia_m	8224	8181	9.81	0.52	12.41	12.91	18.00	2011	2018	2026	2034
Spanish-Argentina_f	7017	7023	5.08	-0.08	6.17	8.91	13.47				
Spanish-Argentina_m	8547	8549	7.60	-0.02	6.19	6.96	11.18	2007	2007	2011	2026
Spanish-Chile_f	4565	4541	15.19	0.52	13.76	15.21	17.82				
Spanish-Chile_m	9371	9383	11.28	-0.13	11.77	12.22	15.62	2007	2024	2046	2069
Turkish-Turkey_f	13035	13044	11.27	-0.07	5.64	10.24	16.16				
Turkish-Turkey_m	27284	27519	11.80	-0.86	4.34	7.30	11.83	2007	2023	2039	2056
All-Average_f	15208	15314	5.98	-0.70	8.51	10.02	13.86				
All-Average_m	33763	34105	2.80	-1.01	5.28	6.11	9.59	2010	2026	2041	2057

AG = Actual growth

DG = Deviation of growth

AV-G = Average growth

Green means sharp increase

Yellow means mild increase

Amber means mild decrease

Red means sharp decrease

5y means 5-year average, i.e., 2016–2020

10y means 10-year average, i.e., 2011–2020

15y means 15-year average, i.e., 2006–2020

Data collected for all language-country combinations (from here on, “cultures” for simplicity) obtained from both methods confirm the existence of a notable gender gap between the scholarly publications of male and female scientists. The gap is observable across all 37 examined countries. Moreover, and perhaps most strikingly, we do not observe any trend that is indicative of the gap narrowing (in terms of absolute total number of publications) in any culture. However, pattern of temporal variations of this gap is distinctly different across cultures. According to the trends presented in Figs 9, 10, 15 and 16 (as well as Figs 11, 12, 17 and 18), three general patterns are differentiable across cultures. These patterns are discussed in the following paragraphs.

The first pattern is a set of cultures in which the gap between total publications of male and female researchers has been exponentially widening over time. This includes almost all Arabic speaking countries (Egypt, Iraq, Jordan, Kuwait, Lebanon, Oman, Qatar, Saudi Arabia, and United Arab Emirates). An exception is Tunisia, whose gap has shown signs of flattening out (or at least slowing down) over the last three to four years (although there is still no discernible sign of narrowing down in a consistent fashion). These are the countries for which the curve of absolute gap (Figs 11, 12, 17 and 18) has a shape of an increasing convex curve, meaning that, not only has the gap been widening every year in these cultures, but also that the gradient of this increase in the gap has been increasing. If the existing trends of these cultures continue, the gender gap will worsen every year, and at an increasing rate. Note that for some of these countries, when we look at the ratios of female to male total productivity (Figs 13, 14, 19 and 20), an extremely slow pattern of increase in the ratio is observed. In these cases, the gender gap is very gradually closing but the discrepancy in number of male- and female-authored publications is increasing due to the rapid overall growth in publications. Take the case of Arabic-Saudi Arabia as an example. When considering the temporal trend in the female to male ratio within this culture, one cannot predict the year when the total research productivity of their female scholars becomes 70% of that of their male scholars (see Tables 2 and 3), even a hundred years into the future. Also, to reach the 50% ratio, the optimistic forecast is more than 60 years from now, while the pessimistic forecast suggests 120 years from now.

The second pattern concerns cultures in which disparity between male and female total productivity is also increasing (similar to that of the countries listed above), except, not at an increasing rate (i.e., exponential way), and rather, at an approximately linear rate. This includes several European countries (e.g., Belgium, Netherlands, Spain, Germany) whose curves of absolute gap have even started to become slightly concave shape, with slight signs of decrease or flattening out in recent years. Less clear examples of this pattern are seen in Italy, Japan, Iran, India and Brazil. These countries are not fast-tracking the closing of the gender gap, but the problem for them does not seem to be exponentially escalating either. As a result, their relative gap has been consistently decreasing and their female to male publication ratios show a more discernible upward trend. For a considerable portion of this cohort of countries, both A-C and A-Z methods predict that, by the middle of the current century they reach a ratio .7 in terms of their female- to male-authored publication (e.g., Italy, Netherlands, Iran, Spain, Brazil). A larger portion of these countries are predicted to reach .5 ratio by the said date.

The third pattern concerns cultures that have managed to maintain a relatively constant absolute gap between total female and male productivity for a sustained amount of time and even have shown small signs of narrowing the gap in the very recent years. This pattern is almost exclusively observable in relation to the developed countries. It is noticeable for Australia, Canada, England, New Zealand, and USA. Both methods (A-C and A-Z) suggest that in all of these countries the absolute gap has had a decrease in 2020 compared to 2019, indicating that this could mark the beginning of a downward trend in gender gap for these countries. However, it is too early to make clear predictions, as no sustained downward trend has been observed in relation to any country yet. According to both sampling methods, the current trends in female to male publication ratios of these countries indicate that by the middle the current century all of them will have reached a ratio of .9.

Focusing on the productivity during 2020 in contrast to the previous trends, striking patterns are observable in relation to male and female productivity. Firstly, both methods suggest that academics of Arabic countries (both genders) have shown the highest degree of actual growth in 2020, compared to their 2019 record of publications, whereas English speaking and some Western European countries have shown the opposite trend. Both male and female academics of Asian countries such as Iran and India have demonstrated positive growth in terms of total productivity, although this is to a lesser degree compared to Arabic countries. This pattern is also observable in relation to South American counties such as Chile, Argentina, Colombia and Mexico.

For most Arabic speaking countries, both sampling methods suggest that female productivity has had a larger growth compared to male total productivity. This includes countries such as Saudi Arabia, Egypt, Kuwait, Lebanon, and Jordan. The only Arabic speaking country for which the growth of total productivity for male academics has been larger than females in 2020 (compared to 2019) is United Arab Emirates. Similarly, when considering the deviation from the projected growth, both methods suggest that female academics of Arabic speaking countries have shown lesser negative deviation from their productivity trend (i.e., their projected productivity) compared to their male counterparts.

When considering English speaking and European countries that have experienced negative impacts on their academic productivity during 2020 compared to previous year(s), the pattern is slightly different. For most of these countries (e.g., Netherlands, USA, England, Canada, Australia, New Zealand) both sampling methods suggest that in terms of actual growth in 2020, female total productivity has been more resistant to the disruptive effects of the pandemic, compared to that of their male counterparts. In certain countries such as USA, Canada and Germany, male total productivity is found to have decreased in 2020 compared to 2019, whereas female total productivity has had positive AG in 2020, and this is confirmed by both sampling methods. However, when the deviation from projected growth is considered, this pattern becomes more mixed. This suggests that that while productivity of female academics seem to have been more resistant to disruptive effects the pandemic compared to male counterparts, their *momentum* has been rather noticeably affected in a comparable manner to that of males in those countries.

Italy shows a growth in publications in 2020, and both sampling methods suggest that the growth has been larger in terms of female productivity. A similar pattern is also observable for Iran, Spain, Chile, Ukraine, Russia, and Brazil. The opposite pattern—i.e., male total productivity showing more resistance to the pandemic effects—is not a common observation in our data (at least when we expect the pattern to have been confirmed by both sampling methods). An exception is India. The productivity of male and female Indian scientists have both increased in 2020, but the amount of increase is estimated to have been larger for male than female, according to both sampling methods.

## Discussion

The findings presented above offer a richly detailed picture of trends in research productivity over the last half-century, as these vary by research field and by author gender. They also allow some tentative inferences about the impact of the Covid-19 pandemic on research productivity based on departures in 2020 from projected trends.

### Trends by research field

The general trends in annual publication numbers are consistently rising across all research fields, a conclusion that is unsurprising given the well documented relentless increase in global research productivity in recent decades. In most fields the rise since 1970 is approximately linear or exponential, with variation in the rate of change from gradual to very steep. Rates tend to be steepest in the more technological fields, such as environmental sciences and engineering disciplines, nanoscience, and geosciences, and also in some Clinical Medicine categories (e.g., Oncology and Health Care Sciences Services). Rises tend to be less steep in the social sciences and humanities disciplines. Nonlinear patterns, such as periods of stasis preceding rapid increases or recent plateaus, are also found in particular cases, and may be interpretable in light of local dynamics in these fields. One challenge facing the interpretations of all of these patterns is the degree to which the extent and timing of publication growth reflects endogenous change in the research fields themselves or changes in the publications indexed by WoS. Nevertheless, the clear pattern of rising global productivity is unambiguous.

### Trends by author gender

The pattern of changes in productivity as a function of author gender, evaluated over a period of 15 rather than 50 years, reflects a similar combination of broad trends and local (country- rather than field-level) variation. The key broad trend is a gradual increase in the proportion of publications with at least one female author, with the ratio of these publications to those with at least one male author increasing from less than 0.4 to more than 0.5 over the study period. The rate of change is troublingly slow, however, with parity very distant and even a 0.7 ratio being forecast as two decades away. In the context of rapid increases in rates of publication, the disparity between the absolute numbers of female- and male-authored publications is growing globally rather than shrinking. Our findings demonstrate the magnitude of the research publication gender gap and the slow but steady rate at which it is narrowing.

### Covid-19 impacts by research field

Inspection of departures of 2020 publication counts from 2019 figures and from long-term polynomial forecasts reveals a general pattern of negative growth that can be cautiously ascribed to the Covid-19 pandemic. However, where research fields are concerned the pattern is relatively weak, with substantial variability across fields. Of the 94 fields examined, a slender majority (51.1%) experienced a reduction in publications in 2020 relative to 2019, with a larger majority (59.6%) falling below forecast. However, many fields did not follow this pattern, some such as the study of infectious diseases and public health showing strong growth for reasons likely to be directly pandemic-related, and others such as environmental science probably as a result of growing attention to climate change issues.

In contrast, the humanities and social sciences (e.g., philosophy, religion, history, political science, education research), were especially hard hit in 2020, showing substantial negative growth in publication counts: on average -12.0% relative to 2019 and an astonishing -37.2% compared to forecast. Negative growth was also common in engineering and computer science fields. The reasons for these negative impacts are not obvious. One possibility is that fields whose primary publication forum is conference proceedings papers, such as computer science, will show a reduction in publications when travel is restricted, and many conferences are suspended. Another is that the traditionally slower peer review processes in the social science and humanities are more impacted by pandemic-related disruptions, resulting in publication delays. These possibilities should be considered in future research, informed by field-specific knowledge. Further studies should also examine whether reduced publication counts in 2020 primarily represent delayed publications that will appear in future years, or whether it represents research that was not conducted due to the pandemic. Whether or not publication counts recover in 2021 and beyond may help to answer this question.

### Covid-19 impacts by author gender

The potentially disproportionate impact of the Covid-19 pandemic on female researchers has been a focus of speculation and some preliminary research, but the present study offers the broadest scope of analysis conducted to date. It suggests that there has been no substantial gender difference in the disruption due to the pandemic. Across 37 national cases, each representing one language-country combination, the average growth in number of female-authored publications from 2019 to 2020 was approximately double the average growth in male-authored publications. Relative to forecast, however, the respective levels of growth are negative to similar degrees. By the A-C method of gender determination, female-authored publications revealed marginally more negative growth than male-authored publications (-1.27% [F] versus -1.14% [M]) but by the alternative A-Z method the pattern reversed (-0.70% [F] versus -1.01% [M]). Although there was notable variability across countries in these patterns, the average pattern is in no way atypical of what is seen in some of the research powerhouses. For example, in the USA, female-authored publications grew from 2019 to 2020 (+3.73% [A-C], +3.28% [A-Z]) while male-authored publications shrank (-1.14% [A-C], -2.03% [A-Z]), and female-authored publications (-1.23% [A-C], -0.86% [A-Z]) fell marginally less below forecast than male-authored publications (-1.42% [A-C], -1.36% [A-Z]). By implication, the Covid-19 pandemic may not have disproportionately disrupted the research productivity of female researchers as has been feared, at least insofar as 2020 publication outputs are concerned. It is possible that disproportionate impacts might emerge at a greater lag, or that they are specific to researchers in particular age or other demographic groups. Future studies should investigate these possibilities.

### Limitations

These gender-related findings must also be considered tentative for two key reasons. First, our method for counting publications as female- and male-authored does not allow direct comparison of numbers of publications because publications with mixed-gender authorship are counted toward both categories. Our method also does not directly represent the relative magnitude of research contributions by female and male researchers because it does not count their fractional contribution to publications but only whether they are categorically present or absent. Second, our novel method for classifying author gender may be imprecise and requires further validation. Encouragingly, two different implementations of the method (A-C and A-Z) generated very similar patterns of findings. It is also important to recognize that although the method may have some error rate, there is no obvious reason to believe it would be systematically biased to allocate author to one gender or another, and it has the significant advantage of identifying author gender at much greater scale than other methods.

### Directions for future research

A potential dimension that was not explored in this work is the investigation of gender gaps within specific research fields. Such investigation can potentially be undertaken using our proposed query-based method. Also, we note that a more representative metric for the investigation of gender productivity gap could perhaps be the frequency of *first authorships* (i.e., counts of male versus female first-authored publications). Not differentiating between ranks of authorships, our results with respect to the effect of Covid-19 pandemic are in contrast with another study [43], for example, who found 19% reduction in 2020 in female first-authorship representation in a sample of medical journals compared to 2019. The current paper considers any publication with mixed gender authorship toward both gender groups. The findings with respect to the effect of the pandemic on genders may as a result show some contrasts to the studies that considered pre-prints [22], those that considered first/last/corresponding authorship [51, 52], or the proportion of representation of male and female authors based on a sample of articles/pre-prints. This further adds to the mixture of evidence that already existed, particularly on the effect of pandemic on male and female productivity (see [53] and [54] for examples) and calls for more nuanced investigations of this problem.

Currently, major scholarly reference databases (e.g., WoS or Scopus) do not offer search options that can differentiate between authorship orders. The detection of author names in such search engines is purely based on a binary determination of whether a name exists within the list of authors, regardless of the position of the author in the list. This places a limitation for the application of the query-based method proposed by this study. However, should such development be implemented by the WoS, then the same query-based method can be readily employed to examine gender disparity based on patterns of first authorships. Given the benefits that such differentiation could bring to academic inquiries of this nature, we recommend that WoS offers this possibility to its users.

A complementary dimension to the analyses presented here is to explore whether country-specific lockdown measures explain the decline in research productivity (similar to the approach of Hipp and Konrad [44] in the context of impact on professional advancement).

It is also important to note that our observations regarding the gender production gap remains limited to overall/gross productivity of male and female scholars and not productivity per individual male or female researchers. The observed gaps are, as such, partly a reflection of more male researcher presence in academia [55, 56]. While the results do speak to an existing and widening gender gap in many geographical regions, they do not have any bearing on whether male scholars on average have been more (or less) productive than their female counterparts. Matters of individual author productivity [46] were beyond the scope of our work.

Also, further research could investigate how the pandemic impacted scholarly productivity across various career stages. However, we do not see a feasible pathway conducting such investigation using query-based methods, as proposed in this work. Traditional methods of sampling published articles or pre-prints as well as self-reported questionnaires could be the pathways for such investigation [55]. An existing study based on analysis of papers published by *Brain*, *Behaviour and Immunity* identifies clear impacts on female first-author representation during 2020 (compared to 2019) as well as a more pronounced impact on female first authorship than last authorship [57]. These existing sets of evidence provide indications that the impact of the pandemic might have been uneven across scholars of various career stages and that early career researchers might have experienced more pronounced setbacks.

On a final note, the effect of any disruption on research activities is often reflected in publications with a time lag. This paper uses publications as the proxy of productivity, which has a lag between work input and publication. It is expected that the setback to academic activity, if any, may become more apparent in publication records during 2021 and onwards. The results presented by this work could at best provide some early indications of these disruptions whereas true effects may only manifest in the coming years. Whether these effects are transient and how long they might last before recovery is to be determined by future research.

## Conclusions

The findings of this study provided an overall picture of quantitative trends of publications in a large sample of research fields. They have several practical implications for research institutions as well as individual researchers. Knowing what research fields are relatively bigger or are expanding faster would, for instance, be of great importance to research institutions when evaluating performances of scholars for matters such as career promotion. This is in consideration of the correlation that exists between the quantity of research articles that are published within a field and the number of citations that researchers of that field receive each year. Similarly, such performance assessments during the pandemic years need to take into account the overall differential impacts that various research fields have endured from the pandemic disruptions. These differential impacts are comprehensively documented in this study.

In relation to gender disparity in overall research production, the findings of this work could guide policy makers who aim to effect changes in long-lasting academic gender gaps. Across different regions of the world, highly differential patterns of gender-related research production gap were unambiguously observable. These observations are particularly important for informing policy makers in countries where the gender gap in research production is not on a tangible path of closing in the foreseeable future, unless effective interventions in academic education, recruitment, research funding allocation, and mentorship are implemented [33, 58]. The findings also exemplify countries that have notably accelerated the closing of their academic gender gap (at least as reflected in the metric of total productivity). This may encourage the exchange of information, experiences, and policy guides between policy makers of these countries and those that seek to intervene with their persistent academic gender gaps. Moreover, while we observed that in countries that endured a larger impact of the pandemic on research productivity, female productivity was often more resilient (e.g., The Netherlands), it should be noted that the effect on the *momentum* of male and female productivity was closely comparable in nearly every case. There was no evidence of any pattern indicating that one gender endured greater impact on its *productivity momentum*.

## Supporting information

S1 Data Data for WoS categories. (ZIP)

S2 Data Data for author gender. (ZIP)
